# Structural visualization of RNA polymerase III transcription machineries

**DOI:** 10.1038/s41421-018-0044-z

**Published:** 2018-07-31

**Authors:** Yan Han, Chunli Yan, Susan Fishbain, Ivaylo Ivanov, Yuan He

**Affiliations:** 1 Department of Molecular Biosciences, Northwestern University, Evanston, IL 60208 USA; 2 Department of Chemistry, Center for Diagnostics and Therapeutics, Georgia State University, Atlanta, GA 30302 USA

## Abstract

RNA polymerase III (Pol III) transcription initiation requires the action of the transcription factor IIIB (TFIIIB) and is highly regulated. Here, we determine the structures of Pol III pre-initiation complexes (PICs) using single particle cryo-electron microscopy (cryo-EM). We observe stable Pol III–TFIIIB complexes using nucleic acid scaffolds mimicking various functional states, in which TFIIIB tightly encircles the upstream promoter DNA. There is an intricate interaction between TFIIIB and Pol III, which stabilizes the winged-helix domains of the C34 subunit of Pol III over the active site cleft. The architecture of Pol III PIC more resembles that of the Pol II PIC than the Pol I PIC. In addition, we also obtain a 3D reconstruction of Pol III in complex with TFIIIB using the elongation complex (EC) scaffold, shedding light on the mechanism of facilitated recycling of Pol III prior to transcription re-initiation.

## Introduction

In eukaryotes, at least three classes of RNA polymerases (Pol I–III) are required for the cellular RNA synthesis^1^. RNA polymerase III (Pol III) transcribes various small stable RNAs that are essential in multiple cellular pathways, including pre-mRNA splicing (U6 snRNA) and protein synthesis (5S rRNA, tRNAs)^2^. Pol III transcription is highly regulated, and elevated Pol III transcript expression is considered a feature of cancer and transformed cells^3–6^.

Pol III transcribes short RNA molecules from three types of promoters. The recruitment and transcription initiation of Pol III on all three types of promoters are dependent on the general transcription factor TFIIIB^7^. In *Saccharomyces cerevisiae*, TFIIIB is composed of three subunits: the TATA box-binding protein (TBP), Brf1 (TFIIB-related factor), and Bdp1 (B double prime). TBP is required for transcription by all three polymerases. While Brf1 shows sequence homology to Rrn7 and TFIIB, which are Pol I and II specific transcription factors, respectively^8^, Bdp1 is unique to Pol III^9^. In addition to their role in Pol III recruitment, Brf1 and Bdp1 have also been shown to function in transcription initiation by mediating transcription bubble opening^10,11^. The initial melting of the promoter DNA requires Bdp1, while Brf1 is responsible for the downstream propagation of the transcription bubble^10^.

Pol III is composed of 17 subunits. The Pol III-specific heterotrimer C82/34/31 locates on top of the C160 clamp^12–14^, and is homologous to the Pol II general transcription factor TFIIE^8^. C82 and C34 both contain multiple winged-helix (WH) domains and regulate Pol III transcription initiation by facilitating the formation of the pre-initiation complex (PIC) through interactions with TFIIIB^15–19^.

Pol III uses a facilitated recycling mechanism to retain itself on the same gene after termination, and the re-initiation of Pol III is a key target of signaling pathways that control cell growth^20–22^. TFIIIB binds the promoter DNA in a very stable manner and possibly remains bound throughout the transcription cycle^20,23^. In addition, Pol III recycling can be efficiently directed by TFIIIB^24^, underlining a role of TFIIIB in both the transcription initiation and re-initiation by Pol III. Therefore, it is of great importance to obtain the structure of Pol III PIC with TFIIIB in order to gain mechanistic insights into the regulation of Pol III transcription.

To this end, we determined the structures of Pol III PICs on a tRNA gene at different functional states using single particle cryo-electron microscopy (cryo-EM). Our PIC structures reveal unique features of the Pol III transcription system and shed light on the mechanism of Pol III promoter opening. During the preparation of our manuscript, Pol III PIC structures assembled on a U6 snRNA gene (SNR6) promoter were published^25,26^. These structures are consistent with our Pol III PIC assembled on the asparagine tRNA tN(GUU)C gene promoter. Importantly, we also obtained a structure of Pol III still engaging with the initiation factor TFIIIB within the gene body, providing a structural platform to understand the full transcription cycle of Pol III-dependent genes.

## Results

### Assembly and cryo-EM reconstruction of Pol III PIC

**Fig. 1:**
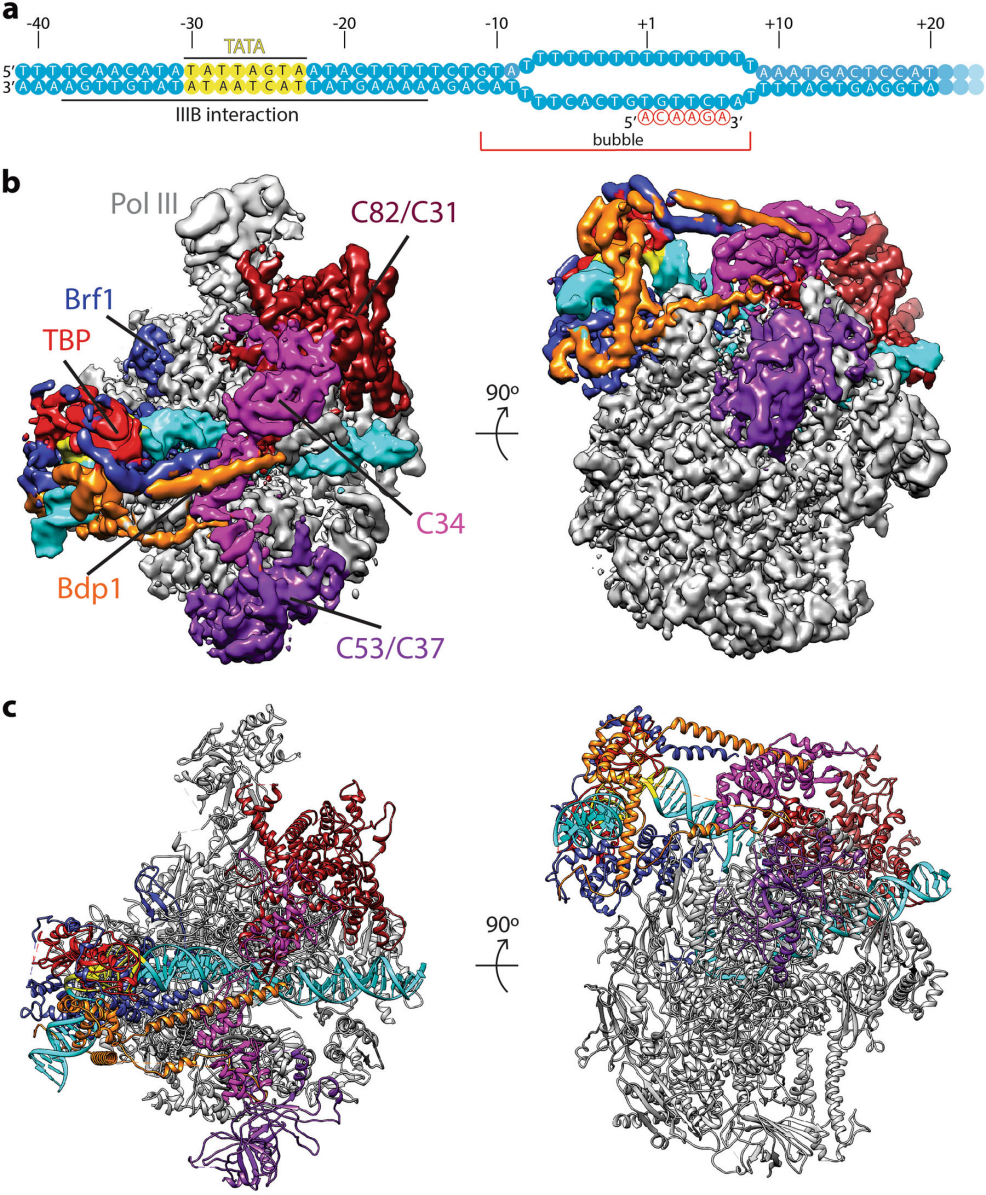
Cryo-EM structure of the Pol III ITC. **a** Nucleic acid scaffold used. The non-template (top) and template (bottom) strands are depicted in cyan. Mismatched sequence is shown as separate strands. RNA is shown in red. TATA box is depicted in yellow. TFIIIB binding region and transcription bubble position are also labeled. **b** Cryo-EM reconstruction of the Pol III ITC. Pol III is colored gray, and the nucleic acid template is colored cyan. The TFIIIB subunits are depicted in blue (Brf1), red (TBP), and orange (Bdp1). Two views, front (left) and bottom (right), are shown. **c** Two views of the Pol III ITC model. Components are colored the same as in **b**

To gain insight into the regulation of Pol III transcription initiation, we assembled the Pol III PIC on an asparagine tRNA tN(GUU)C gene promoter^27^ using purified factors from *Saccharomyces cerevisiae* (Materials and methods; Supplementary Fig. S1). The chimera protein Brf1N-TBPc-Brf1C^28^, which functions in Pol III-dependent transcription both in vitro and in vivo, was used in place of Brf1 and TBP. In order to stabilize the complex, we used a nucleic acid scaffold containing a 16-nucleotide (nt) mismatched transcription bubble in the presence of a 6-nt RNA molecule, mimicking an initial transcribing state (Fig. 1a).

We prepared a cryo sample and obtained a reconstruction of the Pol III initial transcribing complex (ITC) with an overall resolution of 4.1 Å (FSC = 0.143 criterion) (Fig. 1b, c and Supplementary Fig. S2) by single particle analysis using RELION^29,30^. Pol III adopts a conformation similar to that in the elongating Pol III^12^. The reconstruction shows an architecture resembling the Pol II PIC^31–34^. Similar to the Pol II PIC, the Pol III promoter DNA is bent by ~90° after engaging with TBP, a feature that is distinct from the recently resolved Pol I PIC^35–37^. Local resolution estimation shows that the Pol III core region is very rigid with resolution mostly at 3.9 Å, whereas peripheral regions such as the stalk and TFIIIB are more mobile with a lower resolution (5–7 Å) (Supplementary Fig. S2c).

### A complex protein–protein and protein–DNA interaction network within Pol III PIC

**Fig. 2:**
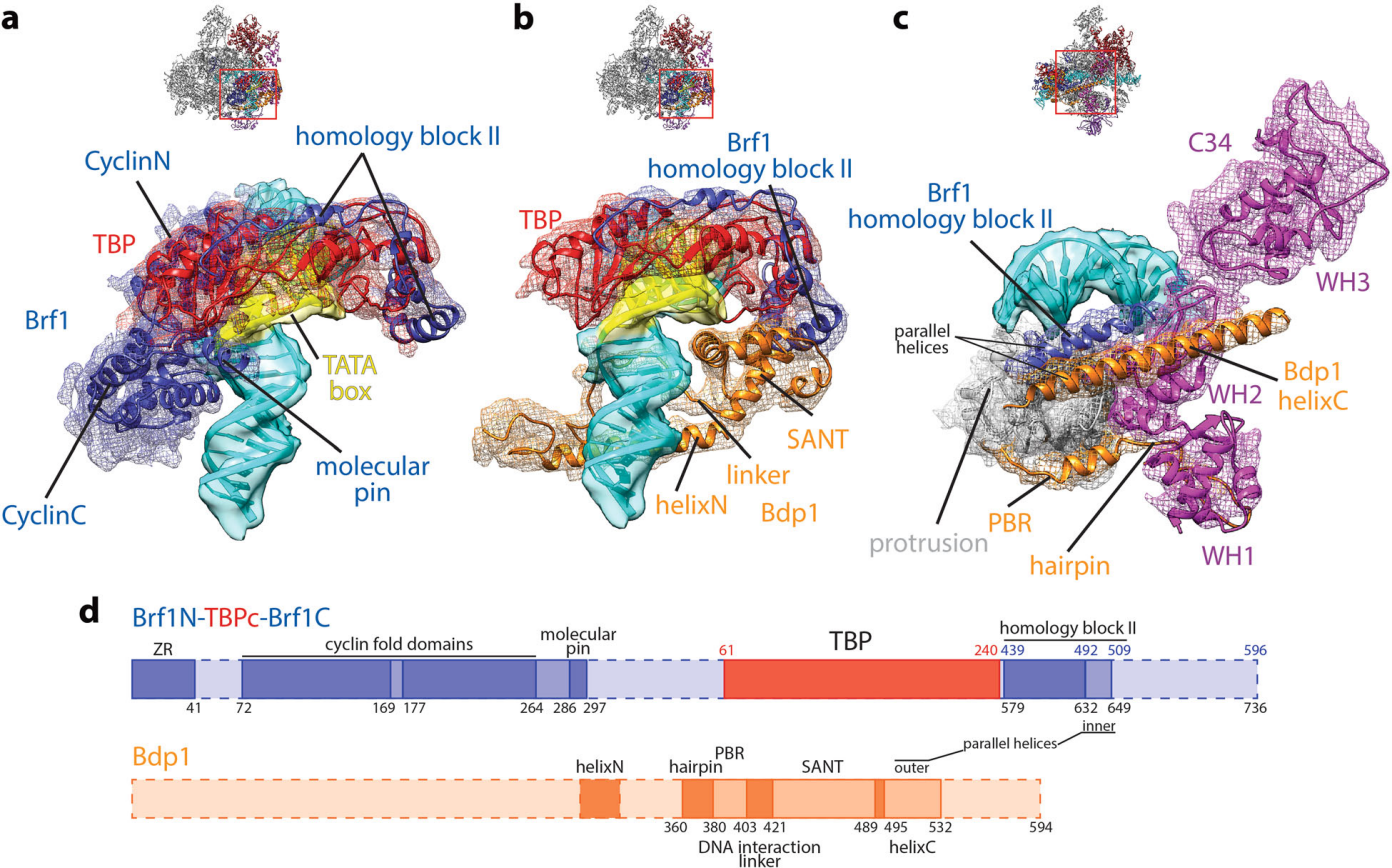
Protein–DNA and protein–protein interactions within the Pol III PIC. **a** The Brf1/TBP/DNA interface. **b** The Bdp1/TBP/DNA interface. **c** The TFIIIB/Pol III interface. Both ribbon diagram and density are shown and are colored as in Fig. 1. Density for DNA is shown as transparent surface, while density for proteins is shown as mesh. Overall views are also shown for each panel, with the same orientation and color scheme. The close-up view is indicated by a red box. Obstructing components are omitted. **d** Schematic representation of TFIIIB components. Dotted outline indicates unresolved regions. The residue numbers for the chimera Brf1N-TBPc-Brf1C protein are labeled below the diagram, whereas the numbers of residues in TBP or Brf1 are labeled above the diagram and colored accordingly

TFIIIB tightly encircles the promoter DNA in the Pol III ITC. On the back side, the two cyclin fold domains of Brf1 clamp the C-terminal stirrup of TBP and interact with promoter DNA both upstream and downstream of the TATA box (Fig. 2a). This interaction is similar to the TFIIB/TBP/DNA complex^38^. Additionally, like the molecular pin in BRF2 (ref. ^39^), the yeast Brf1 contains a similar element, encompassing residues 286–297 (Fig. 2a, d and Supplementary Fig. S3a). This molecular pin lies between the C-terminal cyclin fold domain of Brf1 and TBP, contacting the promoter DNA immediately upstream of the TATA box. It was also observed by Abascal-Palacios et al^25^. On the top, the TBP core domain (residues 61–240) engages the TATA box and bends the promoter DNA by ~90°. In addition, the homology block II of Brf1 (ref.^17^) is also resolved, traversing along the convex surface of TBP (Fig. 2b and Supplementary Fig. S3b), same as observed in the crystal structure of the yeast Brf1/TBP/DNA ternary complex^40^.

In the front, the promoter DNA is contacted by the SANT (Swi3, Ada2, N-Cor, and TFIIIB) domain of Bdp1 (Fig. 2b). The SANT domain also interacts with the N-terminal stirrup of TBP and the homology block II of Brf1 in a similar manner as observed in the human BRF2/TBP/BDP1/DNA complex^41^ (Fig. 2b and Supplementary Fig. S4a). The Brf1 homology block II and the Bdp1 SANT domain together occupy the surface on TBP that is contacted by TFIIA in the Pol II system^42–44^ (Supplementary Fig. S4b). At the bottom of the PIC, two additional structural elements from Bdp1 complete the DNA interaction surface (Fig. 2b). The first is the DNA interaction linker immediately next to the N terminus of the SANT domain (residues 404–421; Fig. 2d). This linker is also observed in the human BRF2/TBP/BDP1/DNA complex^41^, which adopts a different path presumably due to the absence of the polymerase (Supplementary Fig. S4a). It interacts with the minor groove of the DNA at the opposite of the binding site of the C-terminal cyclin fold domain of Brf1 (Fig. 2b), and then turns back and engages the protrusion of Pol III (see below). The second interaction is contributed by a long helix of Bdp1 (helixN, Fig. 2b; residues 293–319; refs.^25,26^).

TFIIIB also forms a complex interaction network with Pol III. Like TFIIB and Rrn7, the N terminus of Brf1 folds into a zinc ribbon (ZR) domain (Supplementary Fig. S5a). The ZR domain contacts the dock of Pol III and connects to a linker region that inserts into the RNA exit channel and interacts with the single-stranded template DNA near the active site. The 6-nt RNA molecule seems to be absent from the active site of Pol III, likely being washed away during sample preparation, or being cleaved by C11 of Pol III. The positioning of Pol III active site by the mismatch bubble at around +7 relative to the TSS (transcription start site) justifies that the initiation complex is in the initial transcribing state. The ZR/polymerase interaction is also observed in Pol I^35–37^ and Pol II^31–34,45,46^ systems, suggesting a conserved role of the ZR domain in TFIIB-like factors. The N-terminal cyclin fold domain of Brf1 contacts Pol III at the wall and protrusion, similar to the Pol II system but distinct from the unique Pol I system (Supplementary Fig. S5).

Another important interface between TFIIIB and Pol III is mediated by the parallel helices within TFIIIB (Fig. 2c, d). The inner helix is the C-terminal helix of Brf1 homology block II (residues 632–649 in the chimera protein; residues 492–509 in full-length Brf1), while the outer helix belongs to Bdp1 at its C terminus (helixC; residues 495–532). Both helices contact and stabilize the second winged-helix (WH2) domain of C34, one subunit of the Pol III-specific heterotrimer C82/34/31 (ref.^8^). This agrees well with the positioning of C34 WH2 domain above the Pol III cleft as probed by chemical crosslinking^14^. In addition, we also observed a small density near the C34 WH3 domain (Supplementary Fig. S6), which could be attributed to the extreme C-terminal end of Brf1 based on crosslinking data^18^. Our structure also shows that the C34 WH2 domain contacts the promoter DNA close to the upstream edge of the transcription bubble (Fig. 2c), suggesting a role of C34 in promoter opening and/or transcription bubble stabilization. Indeed, mutational analyses of C34 WH2 domain showed defective DNA melting during transcription initiation^16^.

Bdp1 also mediates the TFIIIB/Pol III interaction at the protrusion. As mentioned above, the DNA interaction linker immediately on the N-terminal side of the SANT domain interacts with promoter DNA and turns back and wraps around the protrusion (Fig. 2b–d). This protrusion binding region (PBR; residues 381–403) is composed of a linker region and a short α helix. Interestingly, the PBR extends and contacts the C34 WH1 domain, forming a hairpin-like structure (residues 360–380) (Fig. 2c, d). This again agrees with previously reported crosslinking results^47,48^, further emphasizing the role of Bdp1 and C34 in promoter opening.

### Naturally melted promoter DNA using a closed promoter substrate

**Fig. 3:**
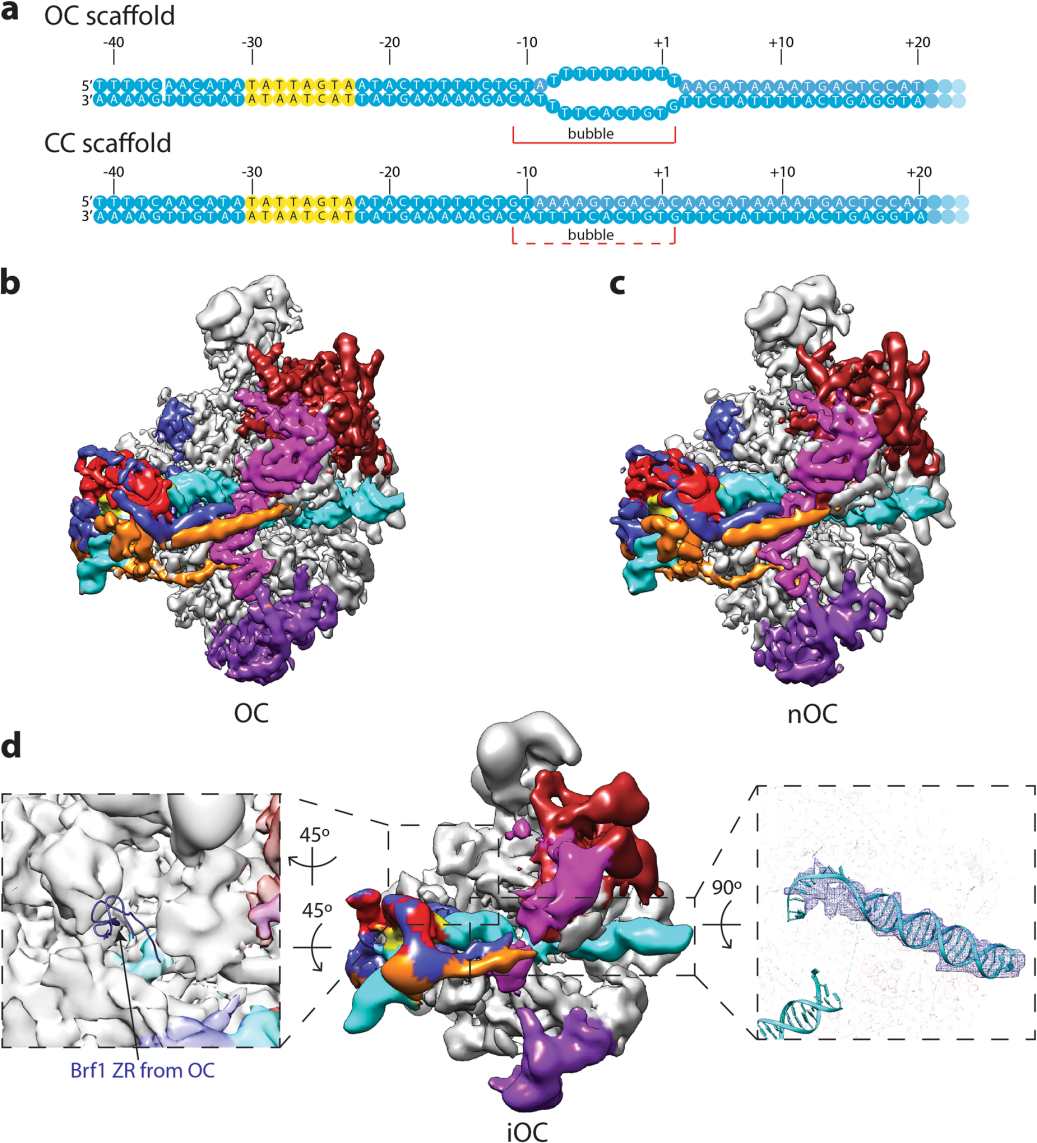
Cryo-EM structures of Pol III open complexes. **a** Nucleic acid scaffold used. Transcription bubble position is also labeled. For the CC (closed complex) scaffold, the bubble is putative, and is labeled using a dashed line. The color scheme is the same as in Fig. 1a. **b, c** Cryo-EM reconstructions of the Pol III OC (**b**) and nOC (natural OC; **c**). The front view is shown, and the color scheme is the same as in Fig. 1b. **d** Cryo-EM reconstruction of the Pol III iOC (intermediate OC). The color scheme is the same as in Fig. 1b. The left zoom in panel shows that the density of the Brf1 ZR domain is absent in the iOC reconstruction. The iOC density is shown as transparent surface, while the Brf1 ZR domain from the OC reconstruction is shown as ribbon diagram. The right zoom in panel shows the comparison of the downstream DNA between OC and iOC. The DNA model from the OC reconstruction is shown as ribbon diagram, and the downstream DNA density in the iOC reconstruction is shown as mesh

To gain insight into the mechanism of Pol III promoter opening, we prepared Pol III PICs using nucleic acid scaffolds mimicking the open complex (OC) and closed complex (CC) (Fig. 3a). The OC scaffold contains a 10-nt mismatched bubble, while the CC scaffold is a fully complementary duplex.

Three-dimensional (3D) refinement yielded a reconstruction at an overall resolution of 4.1 Å for the OC (Fig. 3b and Supplementary Fig. S7), with a similar architecture as the ITC described above, suggesting that Pol III PIC is stable during the OC–ITC transition. Interestingly, the CC dataset gave rise to a reconstruction of an open DNA configuration with an overall resolution of 4.8 Å (Fig. 3c and Supplementary Fig. S8). Albeit at a lower resolution, this structure shows no difference compared to the OC, indicating Pol III PIC can open the promoter DNA efficiently. This spontaneously melted promoter DNA is consistent with previous findings that Pol III promoter opening can be readily achieved at room temperature^49^, the condition under which we assembled the complexes. It was also observed in the recent Pol III OC-PIC structure using the fully duplex SNR6 promoter DNA^25^. We therefore named this OC assembled on the CC DNA scaffold as the nOC (natural OC).

TFIIIB is shown to participate in two steps of promoter opening: Bdp1 is required for the initial melting of the promoter DNA at the upstream end of the transcription bubble, while Brf1 ZR domain is responsible for the downstream propagation of the bubble^10^. For example, the deletion mutants of Bdp1 between residues 355 and 421 can be incorporated into the Pol III PIC, but they fail to mediate transcription bubble opening^10,11^ (Supplementary Fig. S1b, lane 3). We then included these TFIIIB mutants in our complex reconstitution in the hope of capturing a CC or intermediate states. We were able to achieve a 3D reconstruction using Bdp1 Δ355–372 (Fig. 3d and Supplementary Fig. S9). The overall resolution estimated for this map is around 8.5 Å, therefore hindering us from accurately registering the promoter DNA sequence in the map. In addition, in the absence of residues 355–372 of Bdp1, the WH1 domain of C34 becomes disordered, consistent with the contact between the hairpin region of Bdp1 and C34 WH1 domain (Fig. 2c). Interestingly, the reconstruction shows a similar architecture as the OC and ITC described above. First, the downstream DNA is inserted into the active site cleft (Fig. 3d), indicating promoter opening. Although this transcription bubble appears to be less stable than that observed in the OC or ITC, it is somewhat contradictory to the finding that Bdp1 Δ355–372 is defective in promoter opening as probed in footprinting assays^10,11^. This discrepancy could be due to the different promoter sequences used. Another possibility that could also explain the discrepancy is the use of the chimera protein Brf1N-TBPc-Brf1C^25,28^, which generated higher levels of transcription compared to Brf1 and TBP as separate proteins in the absence of TFIIIC^28^. Nevertheless, the observed structural rearrangements of Pol III on the SNR6 promoter^25^ could still occur in the presence of the Bdp1 Δ355–372 to mediate DNA bubble opening.

The second feature of this reconstruction is that the Brf1 ZR domain is flexible and cannot be resolved (Fig. 3d). In line with this, the TFIIIB lobe is extremely flexible, with resolutions ranging from 15 to 25 Å (Supplementary Fig. S9b). Therefore, in addition to the role of Bdp1 in the first step of promoter opening, we speculate that Bdp1 residues 355–421 could also indirectly influence the second step of promoter opening by stabilizing the Brf1 ZR domain in the Pol III PIC. Taken together, the complex assembled using Bdp1 Δ355–372 on the closed DNA scaffold represents an intermediate state right after promoter DNA loading into the active site cleft, hence namely iOC (intermediate OC).

### TFIIIB can associate with Pol III located in the gene body

**Fig. 4:**
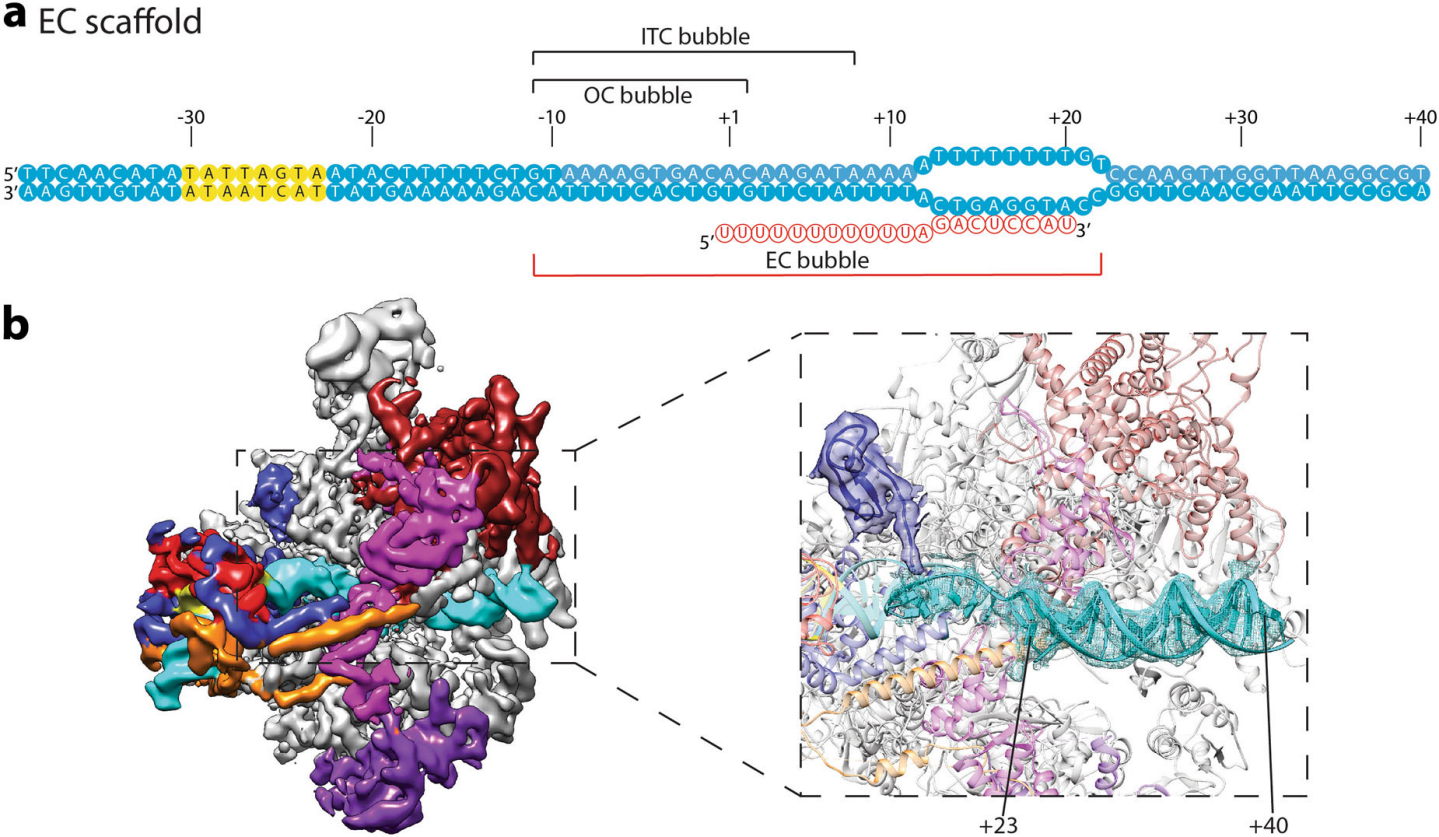
Cryo-EM structure of the Pol III transcription complex assembled on the EC scaffold. **a** Nucleic acid scaffold used. Bubble position in the EC scaffold is labeled under the sequence. Meanwhile, the positions for the OC and ITC bubbles are also indicated above the sequence (see also Figs. 1a and 3a). The color scheme is the same as in Fig. 1a. The scaffold is opened from −11 to +22. **b** Cryo-EM reconstruction of the Pol III transcription complex assembled on the EC scaffold. The color scheme is the same as in Fig. 1b. The zoom in panel shows the fitting of the downstream duplex DNA and the presence of the Brf1 ZR domain in the Pol III transcription complex assembled on the EC scaffold. A total of 18 bp of duplex DNA (from +23 to +40) can be fitted in the downstream DNA density in the Pol III transcription complex assembled on the EC scaffold. DNA density is shown as mesh, and protein density for the Brf1 ZR domain is shown as transparent surface. The rest of the Pol III transcription complex assembled on the EC scaffold is shown as transparent ribbon diagram in the background

We next extended our investigation to the elongation step of Pol III transcription. We designed an elongation bubble from +12 to +22 and annealed a 20-mer RNA molecule to this template to mimic the elongation state (Fig. 4). Using this scaffold, we obtained a 3D reconstruction with an overall resolution of 4.5 Å (Supplementary Fig. S10). To our surprise, TFIIIB still associates with the polymerase. Because the promoter DNA can be spontaneously melted (Fig. 3), one explanation for this reconstruction is that the structure we obtained is still an OC, with the polymerase binding close to the +1 site and occupying the OC bubble (Fig. 4a). However, this is not possible, because a density corresponding to at least 18-bp (base pair) of duplex DNA is clearly visible in the cleft of Pol III, confirming that Pol III actually locates at the designed mismatched bubble (Fig. 4b). If the polymerase was to position at the OC bubble, only 9-bp of duplex DNA would be observed (Fig. 4a; from the downstream edge of the OC bubble +3 to the upstream edge of the mismatch region +11). Polymerase backtracking is not likely to happen either, due to mismatched base pairs at the bubble region. Interestingly, RNA molecule is not seen in the structure. Instead, the Brf1 ZR domain and linker region occupy the RNA exit channel and contact the template strand DNA near the active site (Fig. 4b), similar to the ITC structure (Fig. 1). During active elongation, the extending RNA molecule will displace the Brf1 ZR domain and linker region. We speculate that in the absence of the Brf1 ZR/linker and Pol III interaction, TFIIIB can still associate with both the promoter near the TATA sequence and the polymerase, similar to the iOC structure (Fig. 3d). Therefore, the initiation factor TFIIIB is capable of staying associated with Pol III located in the gene body possibly during the elongation stage, accommodating approximately 33-bp of melted DNA (Fig. 4).

## Discussion

Here, we report the structures of Pol III transcription machinery at different functional states determined using single particle cryo-EM. Our structures suggest a common architecture between Pol II and Pol III PICs, which is distinct from the Pol I PIC. Examinations of the different nucleic acid scaffolds used in our structure determinations reveal unique structural features during Pol III transcription initiation. During the preparation of this manuscript, structures of Pol III PIC assembled on a U6 snRNA gene (SNR6) promoter were published^25,26^. These structures share features with our Pol III PIC assembled on the asparagine tRNA gene promoter. For example, both PICs show an intricate interaction network among TFIIIB, promoter DNA and Pol III; C34 WH1 and WH2 domains are stabilized by TFIIIB near the upstream edge of the transcription bubble on both genes. These shared structural features highly indicate that the mechanisms of Pol III transcription on various types of promoters are likely to be universal. Besides these, some differences between the structures are also worth noting. For example, Abascal-Palacios et al.^25^ observed an extra helix of Bdp1 connecting the helixN (Fig. 2b) in the Clip domain, while Vorländer et al.^26^ and we did not. In addition, using the Bdp1 Δ355–372 mutant, we solved an iOC structure at 8.5 Å resolution (Fig. 3d), while Abascal-Palacios et al.^25^ observed no TFIIIB density on the SNR6 promoter DNA, presumably due to different methods and/or DNA sequences used in the complex assembly reactions. These observations explain why the Bdp1 Δ355–372 mutant shows defects in transcription by destabilizing the Pol III PIC and reemphasize the important roles of Bdp1 residues 355–372 play during Pol III transcription initiation^10,11^. Importantly, we obtained a structure of TFIIIB associating with Pol III using an elongation complex (EC) scaffold, which accommodates a long stretch of DNA (Fig. 4). Interestingly, a TSS scanning mechanism has been proposed for Pol II in yeast^50^, in which Pol II can accommodate long scrunched single-stranded DNA by scanning as long as 100 bp of DNA prior to transcription initiation. Future studies are necessary to provide evidence if these two processes are comparable or utilize distinct mechanisms. Together, these Pol III transcription apparatus structures determined on both the SNR6 promoter^25,26^ and the tRNA gene reported here provide detailed mechanistic insight into Pol III transcription initiation. Additionally, our results also shed new light on the mechanisms of initiation-elongation transition as well as the facilitated recycling of Pol III.

### Initiation

Transcription initiation by all three eukaryotic RNA polymerases is a highly regulated process. For Pol III transcription on tRNA gene promoters (type II), TFIIIB is recruited by TFIIIC, which recognizes the gene’s internal promoter elements^7^. TFIIIB then recruits Pol III, with which it assembles into a PIC and initiates transcription. Unlike Pol II, which requires the ATP hydrolysis activity of TFIIH to open the transcription bubble^31,51–53^, promoter opening of Pol III-dependent genes is ATP-independent. In fact, it has been observed that Pol III-dependent gene promoters can be spontaneously melted simply by incubating the reactions at room temperature^49^. In agreement with this, we obtained an OC structure by assembling the Pol III PIC on a fully duplexed DNA scaffold (nOC; Fig. 3).

**Fig. 5:**
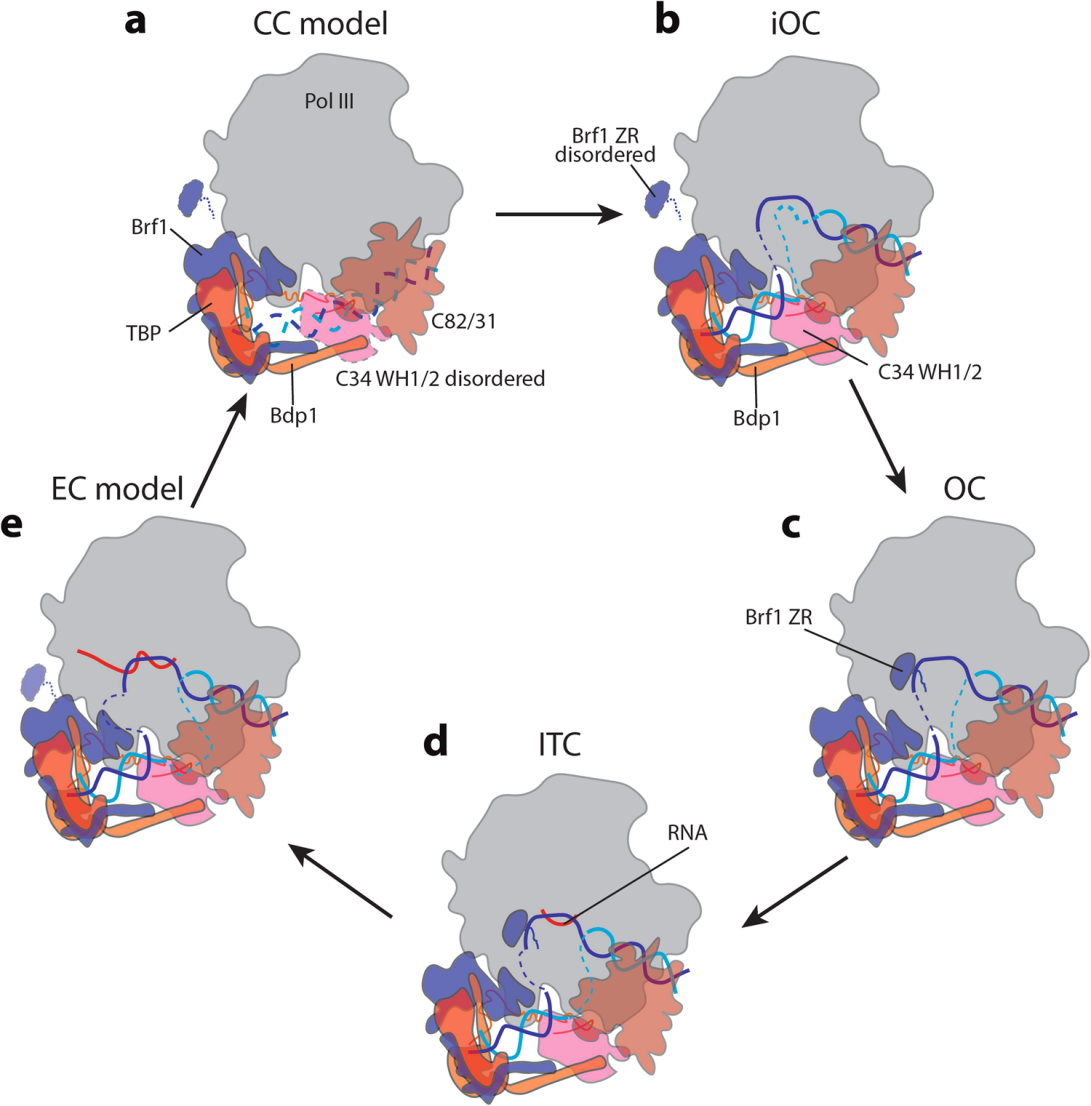
Model for the transcription cycle of Pol III. **a** CC (closed complex) model is proposed based on the PIC structures in this study and a structure of the Pol II CC (PDB ID: 5FZ5)^34^. We propose that in the CC state, the Pol III active site cleft is in an open configuration prior to DNA insertion, and the C34 WH1/2 domains are disordered. **b** Promoter DNA is inserted and starts to melt, forming an iOC. Bdp1 is required for the initial bubble opening. C34 WH1/2 domains become stabilized, keeping the DNA in the active site cleft. In this stage, the Brf1 ZR domain is disordered prior to the fully extension of transcription bubble. **c** Following the iOC, the Brf1 ZR domain is stabilized in the RNA exit channel, and extends the transcription bubble, forming an OC. **d** Once the OC is formed, Pol III starts to synthesize RNA, entering the ITC state. Our structures of OC and ITC look alike, suggesting that Pol III PIC is primed to transcription initiation at an early stage. **e** Pol III continues to synthesize RNA and enters the elongation state. During this process, TFIIIB stays bound with Pol III, while the Brf1 ZR domain needs to be displaced. Because Pol III-transcribed genes are typically very short, this could be the mechanism for the facilitated recycling of Pol III, giving rise to a very efficient transcription output. Coloring of the components is the same as in Fig. 1, except that the template DNA is shown in blue and the non-template DNA is shown in cyan. Dotted outlines of protein components indicate that the corresponding protein is disordered or flexible. The transcription bubble is depicted as dashed blue and cyan lines, with growing sizes through the transcription cycle to indicate the progression of Pol III

The spontaneous opening of the Pol III-dependent promoter DNA is carried out in two steps that require Bdp1 and Brf1 (ref. ^10^). In addition, the C34 WH2 domain is also shown to be important for promoter opening^16^. Based on these data and our structural analyses, we propose a model for Pol III transcription initiation (Fig. 5). Upon recruitment to the promoter, the Pol III active site cleft is in an open configuration^12^, ready for the insertion of the DNA (Fig. 5a). TFIIIB may occupy a slightly different location to allow insertion of the DNA into the cleft of Pol III, when compared with the Pol II system (Supplementary Fig. S11). Once promoter DNA is inserted, Pol III cleft closes. Meanwhile TFIIIB stabilizes C34 WH domains over the cleft, presumably keeping the DNA from escaping the cleft and also inducing the initial melting of the promoter DNA near position −10 (Fig. 5b). This could be a semi-stable state similar to the reconstruction we solved using the Bdp1 Δ355–372 mutant (Fig. 3d), as the bubble opening is a reversible process^49^. Next, the Brf1 ZR domain is stabilized near the Pol III dock and the linker region inserts into the RNA exit channel to contact the promoter DNA in the active site cleft, facilitating the extension of the transcription bubble (Fig. 5c). Bdp1 residues 355–421 and/or C34 are necessary to stabilize the Brf1 ZR domain (Fig. 3d). When the bubble is fully extended, Pol III will start synthesizing RNA and enter the ITC state (Fig. 5d). Our structures show that Pol III OC resembles ITC, suggesting that Pol III PIC is primed to initiate transcription at an early step of PIC formation.

### Elongation and re-initiation

One of the distinct features of Pol III transcription is the facilitated recycling of Pol III onto the same gene promoter, directing multiple rounds of transcription^20^. TFIIIB is responsible for the recycling of Pol III in vitro^24^. In line with this, we observed in our cryo-EM reconstruction that TFIIIB stably associates with Pol III in the elongating mode positioned further downstream into the gene body (Fig. 4). Given the fact that Pol III-dependent genes are relatively short, we propose that promoter-bound TFIIIB stays engaged with Pol III throughout the transcription cycle. We therefore extend the model to include the elongation and re-initiation steps (Fig. 5e). As the RNA is extended, Pol III moves downstream into the gene body, accommodating a long stretch of melted DNA, between TFIIIB and Pol III (Fig. 5e). C34 WH1 and WH2 domains are also stabilized over the cleft, consistent`with the recent report that mutations in the tandem WH domains of C34 reduce transcription elongation efficiency^54^. During the RNA extension step, the ZR domain and linker region of Brf1 need to be displaced, which may be achieved by the extending nascent RNA. When reaching the termination signal, consisting of typically 5 to 7T residues^55^, Pol III will be released from the DNA (Fig. 5e), which involves the flexible linker within C37 (ref. ^12^). Subsequently, the released polymerase could be transferred back to the promoter as TFIIIB bridges the promoter with Pol III throughout the transcription cycle (Fig. 5). This is consistent with the finding that TFIIIC and/or TFIIIA are dispensable for the re-initiation of Pol III, whereas TFIIIB is required^23^. Future structural studies will be necessary to reveal the detailed molecular mechanisms during this process.

## Materials and methods

### Protein purification

#### Pol III and TFIIIC

Pol III was purified from a yeast strain containing a TAP tag at the C terminus of subunit C53 (GE Dharmacon, YSC1178-202230266), and TFIIIC was purified from a yeast strain containing a TAP tag at the C terminus of subunit Tfc8 (GE Dharmacon, YSC1178-202233621). Tandem affinity purification was performed as following. Twelve liters of yeast were grown to an optical density at 600 nm (OD_600_) of 4–5 in YPD (3% glucose). Next, cells were harvested by centrifugation and washed with 200 ml of cold TAP Extraction Buffer (40 mM Tris pH 8, 250 mM ammonium sulfate, 1 mM EDTA, 10% glycerol, 0.1% Tween-20, 5 mM dithiothreitol [DTT], 2 mM phenylmethylsulfonyl fluoride [PMSF], 0.31 mg/ml benzamidine, 0.3 μg/ml leupeptin, 1.4 μg/ml pepstatin, 2 μg/ml chymostatin). Cells were resuspended in 150 ml cold TAP Extraction Buffer and lysed in a BeadBeater (Biospec Products). Cell debris was removed by centrifugation at 14,000 × *g* at 4 °C for 1 h. For the first affinity step, 2 ml IgG Sepharose beads (GE Healthcare) were incubated with the lysate at 4 °C overnight. The beads were next washed and resuspended in 4 ml cold TEV (tobacco etch virus) Cleavage Buffer (10 mM Tris pH 8, 150 mM NaCl, 0.1% NP-40, 0.5 mM EDTA, 10% glycerol). TEV cleavage using 25 µg of TEV protease was performed at room temperature for 1 h with gentle shaking. The TEV protease-cleaved products were collected, and the IgG beads were washed with three column volumes (~6 ml total) cold Calmodulin Binding Buffer (15 mM HEPES pH 7.6, 1 mM magnesium acetate, 1 mM imidazole, 2 mM CaCl_2_, 0.1% NP-40, 10% glycerol, 200 mM ammonium sulfate, 5 mM DTT, 2 mM PMSF, 0.31 mg/ml benzamidine, 0.3 μg/ml leupeptin, 1.4 μg/ml pepstatin, 2 μg/ml chymostatin). CaCl_2_ was added to the combined eluate at a final concentration of 2 mM and incubated with 0.8 ml Calmodulin Affinity Resin (Agilent Technologies) at 4 °C for 2 h. After incubation, the beads were washed with cold Calmodulin Binding Buffer and cold Calmodulin Wash Buffer (same as Calmodulin Binding Buffer, but containing 0.01% NP-40), and bound proteins were eluted in 11 0.4 ml fractions with Calmodulin Elution Buffer (15 mM HEPES pH 7.6, 1 mM magnesium acetate, 1 mM imidazole, 2 mM EGTA, 10% glycerol, 0.01% NP-40, 200 mM ammonium sulfate) at room temperature.

#### Bdp1

Yeast Bdp1 was PCR amplified from genomic DNA and cloned into pET21b (+) using restriction sites *Sal*I and *Not*I, resulting in a 6× His tag at the C terminus of Bdp1. The construct was then transformed into *Escherichia coli*-competent cells BL21 (DE3) harboring the plasmid pRARE for rare codon expression. Bdp1 expression and purification were performed as described for B″ (138–594)^56^ with modifications. Briefly, 1 l transformed cells were grown in LB at 37 °C to an OD_600_ of 0.4, and the cells were then induced using 1 mM isopropyl β-d-1-thiogalactopyranoside (IPTG) for 2 h at 37 °C. Cells were harvested by centrifugation and washed with a buffer containing 20 mM Tris-HCl pH 7.5 and 150 mM NaCl. Cell pellets were flash frozen in liquid nitrogen and stored at −80 °C before lysing. For lysis, the cell pellet was first thawed on ice, and then was resuspended in 1 ml of Bdp1 Lysis Buffer 1 (50 mM Tris pH 8, 0.1 mM EDTA, 5% glycerol, 10 mM β-mercaptoethanol [BME], 0.5 mM PMSF, 1 µg/ml pepstatin, 1 µg/ml leupeptin, 300 µg/ml lysozyme) per gram of pellet. The cell suspension was incubated on ice for 30 min, followed by addition of Tween-20 for a final concentration of 0.1%. The cell suspension was then sonicated five times for 30 s each on ice, with at least 1 min rest time. The lysate was diluted two-fold using Bdp1 Lysis Buffer 2 (50 mM Tris pH 8, 1 M NaCl, 5% glycerol, 10 mM BME, 0.5 mM PMSF, 1 µg/ml pepstatin, 1 µg/ml leupeptin), and sonicated again as above. The lysate was then cleared by centrifugation at 12,100 rpm 4 °C for 1 h, followed by addition of MgCl_2_ and imidazole to a final concentration of 7 and 10 mM respectively to the supernatant. The cleared lysate was then passed through 500 µl of HIS-Select Nickel Affinity Gel (Sigma-Aldrich) equilibrated with Bdp1 Binding Buffer (20 mM HEPES pH 7.6, 500 mM NaCl, 7 mM MgCl_2_, 0.01% Tween-20, 10% glycerol) in a gravity column. The column was then washed with three 0.5 ml of Bdp1 Binding Buffer + 10 mM imidazole + 10 mM BME + 0.5 mM PMSF, and four 1 ml of Bdp1 Binding Buffer + 30 mM imidazole + 10 mM BME + 0.5 mM PMSF. Bound protein was eluted with two 1 ml of Binding Buffer + 60 mM imidazole + 10 mM BME + 0.5 mM PMSF, and two 1 ml of Binding Buffer + 250 mM imidazole + 10 mM BME + 0.5 mM PMSF. Bdp1 was eluted in fractions with 30 and 60 mM imidazole. Fractions containing Bdp1 were then combined and the salt concentration was diluted to 100 mM using Buffer 0 (20 mM HEPES pH 7.6, 7 mM MgCl_2_, 0.01% Tween-20, 10% glycerol), and Bdp1 was further purified using SP Sepharose (GE) in batch mode. Bdp1 was eluted using Buffer 500 (Buffer 0 + 500 mM NaCl), followed by dialysis against Dialysis Buffer (20 mM HEPES pH 7.6, 100 mM ammonium sulfate, 7 mM MgCl_2_, 0.01% Tween-20, 10% glycerol, 0.2 mM PMSF, 5 mM DTT) and concentration. Bdp1 Δ355–372 was cloned by quick change mutagenesis and was purified as the wild-type protein.

#### Brf1N-TBPc-Brf1C

The chimera protein Brf1N-TBPc-Brf1C^28^ was cloned into pET21b (+) as following. First, full-length Brf1 was PCR amplified from genomic DNA and cloned into pET21b (+) at restriction sites *Sal*I and *Not*I, resulting in a 6× His tag at the C terminus of Brf1. TBPc was PCR amplified from genomic DNA using primers 5′- TCTGAAAACGAAACAAGGAAACGCAAACTTTCTGAAGCGATGCCATGGTCAGGTATTGTTCCAACACTAC-3′ and 5′-TGTGTCCGTTGTGGGTAACAAGTGTAAATTGCGAGGACTGCCCGATCCACTACCTGACCCGGATCCACTGCCCATTTTTCTAAATTCACTTAGCAC-3′. This piece of amplified DNA was then inserted into pET21b-Brf1 by quick change, to replace residues 383–438 of Brf1. DNA sequence was confirmed by sequencing. Brf1N-TBPc-Brf1C was transformed into BL21 (DE3) pRARE, and purified as described^28^ with modifications. Briefly, 2 l transformed cells were grown in LB at 37 °C to an OD_600_ of 0.5–0.8, and the cells were then induced using 0.5 mM IPTG for overnight at 18 °C. Cells were harvested by centrifugation and washed with a buffer containing 20 mM Tris-HCl pH 7.5 and 150 mM NaCl. Cell pellets were flash frozen in liquid nitrogen and stored at −80 °C before lysing. For lysis, the cell pellet was first thawed on ice, and then was resuspended in 35 ml of BT Lysis Buffer (20 mM HEPES pH 7.6, 25 μM EDTA, 1.14 M NaCl, 5% glycerol, 10 mM BME, 0.5 mM PMSF, 1 µg/ml leupeptin, 1 µg/ml pepstatin, 300 µg/ml lysozyme), and incubated on ice for 1 h, followed by sonication. Lysate was clarified by centrifugation at 13,000 × *g* 4 °C for 1 h. The cleared lysate was then passed through 500 µl of HIS-Select Nickel Affinity Gel (Sigma-Aldrich) equilibrated with BT Lysis Buffer without BME or lysozyme in a gravity column. The column was then washed with five 1 ml of BT Wash Buffer 1 (20 mM HEPES pH 7.6, 7 mM MgCl_2_, 0.5 M NaCl, 10 mM imidazole, 5% glycerol, 10 mM BME, 0.5 mM PMSF, 1 µg/ml leupeptin, 1 µg/ml pepstatin) and five 1 ml of BT Wash Buffer 2 (20 mM HEPES pH 7.6, 7 mM MgCl_2_, 0.5 M NaCl, 20 mM imidazole, 5% glycerol, 10 mM BME, 0.5 mM PMSF, 1 µg/ml leupeptin, 1 µg/ml pepstatin). Protein was eluted by five 1 ml of BT Elution Buffer (20 mM HEPES pH 7.6, 7 mM MgCl_2_, 0.5 M NaCl, 200 mM imidazole, 5% glycerol, 10 mM BME, 0.5 mM PMSF, 1 µg/ml leupeptin, 1 µg/ml pepstatin). The chimera protein was eluted in both BT Wash Buffer 2 and BT Elution Buffer. Fractions containing the chimera protein was pooled and the salt concentration was diluted to 100 mM using Buffer 0. Brf1N-TBPc-Brf1C was further purified using SP Sepharose (GE) in batch and dialyzed against Dialysis Buffer and concentrated.

### In vitro transcription assay

Two microliters of purified Pol III (~1 µM) was incubated in the presence of approximately 2 molar excess of recombinant Brf1N-TBPc-Brf1C, recombinant Bdp1, and TAP purified TFIIIC for 15 min at room temperature in 1× TB buffer (12 mM HEPES pH 7.9, 0.12 mM EDTA, 12% glycerol, 8.25 mM MgCl_2_, 60 mM KCl, 1 mM DTT, 0.05% NP-40, 100 µM ZnSO_4_, and 1 µl rRNasin Plus [Promega]) at a total volume of ~17 µl. A template consisting of 2 µl of a purified PCR product (~100 ng) containing the asparagine tRNA tN(GUU)C gene (−47 to +100) was added to the reaction and incubated for 5 min at room temperature. Five millimolar ribonucleotides (NTPs) were added to the reaction and incubated for 1 h at room temperature. Subsequently, 1 µl 10× DNaseI reaction buffer and 1 µl DNaseI (New England Biolabs) were added and incubated for 5 min at 37 °C, followed by addition of 1 µl Proteinase K (New England Biolabs) and incubation for another 5 min at 37 °C. The reaction was stopped by adding 4 µl 6× DNA loading dye (Thermo Fisher Scientific) and then run on a 10% TBE-7 M urea gel. The gel was stained using SYBR Gold (Thermo Fisher Scientific) and visualized on a UV gel imager.

### Scaffold preparation

Oligonucleotides for assembling the promoter scaffold were purchased from Integrated DNA Technology (IDT). The sequence is based on the Asn tRNA tN(GUU)C. Oligonucleotides used are: non-template strand, 5′-CAACTTGGCCATGGAGTCATTTTATCTTGTGTCACTTTTACAGAAAAAGTATTACTAATATATGTTGAAAAACTGGGGAATTCCATGGTCCGTA-3′; CC template strand, 5′-TTTTCAACATATATTAGTAATACTTTTTCTGTAAAAGTGACACAAGATAAAATGACTCCATGGCCAAGTTG-3′; OC template strand, 5′-TTTTCAACATATATTAGTAATACTTTTTCTGTATTTTTTTTTTAAGATAAAATGACTCCATGGCCAAGTTG-3′; ITC template strand, 5′-TTTTCAACATATATTAGTAATACTTTTTCTGTATTTTTTTTTTTTTTTTAAATGACTCCATGGCCAAGTTG-3′; EC non-template strand, 5′-ACGCCTTAACCAACTTGGCCATGGAGTCATTTTATCTTGTGTCACTTTTACAGAAAAAGTATTACTAATATATGTTGAAAAACTGGGGAATTCCATGGTCCGTA-3’; EC template strand, 5′-TTCAACATATATTAGTAATACTTTTTCTGTAAAAGTGACACAAGATAAAAATTTTTTTTGTCCAAGTTGGTTAAGGCGT-3′; ITC RNA, 5′-ACAAGA-3′; EC RNA, 5′-UUUUUUUUUUUAGACUCCAU-3′. All scaffolds were also annealed with biotin-5′-TACGGACCATGGAATTCCCCAGT-3′ for complex purification. Lyophilized DNA oligos were first resuspended in ultra-pure water for a final concentration of 100 µM. Assembly of the template used for EM studies was done by mixing template strand and non-template strand oligos with a 1:1 molar ratio at a final concentration of 10 µM in ultra-pure water, and denaturing in boiling water bath for 5 min, followed by gradually cooling down to room temperature in 2 h. For scaffold containing RNA molecules, RNA and annealed DNA template was first mixed with a 1.5:1 molar ratio and heated in a water bath at 45 °C for 5 min, followed by gradually cooling down to room temperature. Assembled nucleic acid templates were subsequently diluted to 2 µM concentration using ultra-pure water.

### Pol III PIC assembly

To assemble the Pol III PICs for negative stain EM analysis, 2 µl of purified yeast Pol III (~1 µM) was first mixed 1:1 with assembly buffer (12 mM HEPES pH 7.9, 0.12 mM EDTA, 12% glycerol, 8.25 mM MgCl_2_, 1 mM DTT, 2.5 ng/µl dI–dC, 5 µM ZnCl_2_, and 0.05% NP-40 [Roche]), and incubated with 0.25 µl of the biotinylated nucleic acid template (2 µM) for 5 min at room temperature. Next, Brf1N-TBPc-Brf1C and Bdp1 were added to the mixture at an approximate 2:1 molar ration to Pol III and incubated for 5 and 10 min, respectively. Assembled complex was immobilized onto the magnetic streptavidin T1 beads (Invitrogen) which had been equilibrated with the assembly buffer plus 90 mM ammonium sulfate. Following washing of the beads two times using a washing buffer (10 mM HEPES, 10 mM Tris, pH 7.9, 5% glycerol, 5 mM MgCl_2_, 50 mM KCl, 1 mM DTT, 0.05% NP-40, 5 µM ZnCl_2_), the complex was eluted by incubating the beads at room temperature for 45 min with 3 µl digestion buffer containing 10 mM HEPES, pH 7.9, 10 mM MgCl_2_, 50 mM KCl, 1 mM DTT, 5% glycerol, 0.05% NP-40, 0.5 unit/µl *Eco*RI-HF (New England Biolabs).

Samples used for cryo-EM were prepared similarly to the negative stained samples, but with the following modifications. To prepare Pol III OC and nOC for cryo-EM analysis, 30-fold more material than a single negative stain sample was used. In addition, the beads were washed one more time. For complex elution, the digestion buffer contained only 2.5% glycerol and 2 unit/µl *Eco*RI-HF, and 4 µl was used. The reaction was incubated at 37 °C for 1 h. Pol III ITC and the complex using the EC scaffold were prepared similarly, but with 40- and 10-fold the material of a single negative stain sample, respectively. To prepare the Pol III PICs with Bdp1 Δ355–372 for cryo-EM analysis, 10-fold the material of a single negative stain sample was used. Pol III was first incubated with the nucleic acid scaffold for 5 min at room temperature, followed by addition of assembly buffer plus 60 mM KCl and the incubation was extended for 10 min.

### Electron microscopy

Negative stain sample preparation and data collection were performed as previously described^36^.

Preparation of the Pol III PICs for cryo-EM analysis was performed as previously described^36^. Briefly, the eluted complex was first briefly crosslinked using 0.19% glutaraldehyde on ice and under very low illumination conditions for 5 min. The sample (~4 µl) was then immediately loaded onto a 400 mesh C-flat grid containing 4 µm holes with 2 µm spacing (C-flat 4/2; Electron Microscopy Sciences). A thin carbon film was floated onto the grid before it was plasma cleaned for 10 s at 5 W power using a Solarus plasma cleaner (Gatan) equipped with air immediately before sample deposition. The grid was incubated with the sample at 4 °C and 100% humidity in a Vitrobot (FEI) under low illumination conditions, before blotted for 4 s at 25 force and plunge-frozen in liquid ethane. The frozen grids were stored in liquid nitrogen until imaging.

Cryo-EM data collection was performed using a JEOL3200FS transmission electron microscope (JEOL) equipped with a K2 Summit direct electron detector (Gatan) operating at 300 kV. For ITC and nOC, data were acquired with the K2 camera operating in superresolution mode at a nominal magnification of ×30,000 (0.59 Å per pixel), using a range of defocus values (from −1.5 to −4.5 μm). In total, 2019 and 1760 movie series, for ITC and nOC respectively, were collected using the Leginon data collection software^57^. Forty-frame exposures were taken at 0.3 s per frame (12 s total exposure time), using a dose rate of 2 *e*^−^ per pixel per second, corresponding to a total dose of 68.9 *e*^−^ Å^−2^ per movie series. For OC and Pol III transcription complex assembled on the EC scaffold, data were collected using the K2 camera in counting mode at a nominal magnification of ×30,000 (1.18 Å per pixel). in all, 1352 movie series for OC with defocus values ranging from −1 to 3.5 µm and 2317 movies series for the Pol III transcription complex assembled on the EC scaffold with defocus values ranging from −1.5 to −4.5 µm were collected using Leginon. As described above, 40-frame exposures were taken at 0.3 s per frame (12 s total), using a dose rate of 8 *e*^−^ per pixel per second, corresponding to a total dose of 68.9 *e*^−^ Å^−2^ per movie series. For PIC using Bdp1 Δ355–372, data were collected on the JEOL3200FS operating at 200 kV and the K2 camera in counting mode at a nominal magnification of ×30,000 (1.115 Å per pixel). In all, 1055 movie series with defocus values ranging from −1 to −4.5 µm were acquired. Similarly, 40-frame exposures were taken at 0.3 s per frame (12 s total exposure time), using a dose rate of 8 *e*^−^ per pixel per second, corresponding to a total dose of 77.2 *e*^−^ Å^−2^ per movie series.

### Image processing and 3D reconstruction

Negative stain data pre-processing was performed using the Appion processing environment^58^. Particles were automatically selected from the micrographs using a difference of Gaussians (DoG) particle picker^59^. The contract transfer function (CTF) of each micrograph was estimated using CTFFind4 (ref.^60^), the phases were flipped using CTFFind4, and particle stacks were extracted using a box size of 96 × 96 pixels. Two-dimensional classification was conducted using iterative multivariate statistical analysis and multi-reference alignment analysis (MSA-MRA) within the IMAGIC software^61^. 3D reconstruction of negative stained data was performed using an iterative multi-reference projection-matching approach containing libraries from the EMAN2 software package^62^. The cryo-EM map of the apo Pol III (EMD-3179)^12^ was low-pass filtered to 30 Å, which was used as the initial model for the reconstruction of the negatively stained Pol III PIC samples.

Cryo-EM data were pre-processed as follows. Movie frames were aligned and electron dose was weighted using MotionCor2 (ref.^63^) to correct for specimen motion. Movies collected under superresolution mode were binned by 2 during motion correction. Particles were automatically selected from the aligned and dose-weighted micrographs using Gautomatch (developed by Kai Zhang, MRC Laboratory of Molecular Biology, Cambridge, UK). The CTF of each micrograph and of each particle was estimated using Gctf^64^. All 3D classification and refinement steps were performed within RELION 2.1 (ref.^30^).

For OC, ITC, and the Pol III transcription complex assembled on the EC scaffold, the initial set of 242,128, 365,148, and 318,508 particles, respectively, was binned by 2 and subjected to an initial 3D auto-refinement, using the negative stain reconstruction of the complex low-pass filtered to 30 Å as the initial reference. Subsequently, a 3D classification was performed on the picked particles using the 3D auto-refined model low-pass filtered to 30 Å as the initial reference. For OC, two out of five classes (classes 4 and 5) in this classification, corresponding to 38,656 and 35,652 particles (Supplementary Fig. S7b), were indicative of well-preserved complex with sharp structural features and were selected for further processing. These two classes were combined and then unbinned and subjected to 3D auto-refinement within RELION, yielding a structure at an average resolution of 4.1 Å after post-processing (Supplementary Fig. S7). All resolutions reported herein correspond to the gold-standard Fourier shell correlation (FSC) using the 0.143 criterion^65^. Local resolution estimation indicated that the density for TFIIIB was at lower resolution than Pol III (Supplementary Fig. S7b). Subsequently, soft masks were applied around the TFIIIB density during further 3D refinement within RELION. This procedure resulted in an improved reconstruction of TFIIIB, with an overall resolution of 4.7 Å (Supplementary Fig. S7). For ITC, the 3D classification after auto-refinement yielded one class (class 5, 28.3% of total particle) (Supplementary Fig. S2c) with well-preserved structural features. Further unbinning and refinement of this class revealed a 3D reconstruction at an average resolution of 4.1 Å (Supplementary Fig. S2). The Pol III transcription complex assembled on the EC scaffold is similar to the ITC, with one class containing 14.8% of the particles revealing a 3D reconstruction at an average resolution of 4.5 Å (Supplementary Fig. S10).

For nOC, the initial set of 303,453 particles was binned by 2 and subject to an initial 3D classification with alignment using the negative stain reconstruction of the complex low-pass filtered to 30 Å as the initial reference. Class 1 (32% of the total particles) contains the full complex. Particles in this class were unbinned, and further refinement of the particles in class 1 revealed a 3D reconstruction at an average resolution of 4.8 Å (Supplementary Fig. S8). For iOC, the initial set of 119,415 particles were processed similarly to the nOC, except that the particles were binned by 2 throughout data processing. One class (class 2, 29.3% of the total particles) yielded a reconstruction at an overall resolution of 8.5 Å (Supplementary Fig. S9)

The final density maps were automatically sharpened using the post-processing program within RELION and then filtered according to local resolution estimated within RELION 2.0. Volume segmentation, automatic rigid-body docking, figure, and movie generation were performed using UCSF Chimera^66,67^.

### Model building

To build the structure of the Pol III PIC, we used the known structures of elongating Pol III (Pol III EC) (PDB ID: 5FJ8)^12^, the yeast Brf1/TBP/DNA crystal structure (PDB: 1NGM)^40^, and the human crystal structure of a TFIIIB/DNA complex (PDB ID: 5N9G)^41^ as starting points. To model downstream DNA, the DNA structure from a previous model of the yeast Pol III EC (PDB ID: 5FJ8) was fitted into the density and modified to include the DNA bubble region. The upstream DNA was built from the human TFIIIB/DNA complex and was rigid-body fitted into the density.

To model the yeast Bdp1 SANT domain (residues 419–477), the crystal structure of the human Bdp1 (PDB ID: 5N9G)^41^ was used as a template to construct the yeast Bdp1 structure. Two additional helixes were built in the C terminus based on the EM density and secondary structure prediction. N terminus regions of Bdp1 were built manually using information from secondary structure prediction^68^, observed photo-crosslinking (K281 of Bdp1 crosslinked to Brf1)^47^, and less stable density in the iOC reconstruction using Bdp1 Δ355–372 (Supplementary Fig. S9d).

The homology model of the Brf1 cyclin fold domains (residues 71–265) was built using MODELER 9V15 software^69^ based on the alignments with the archaeal TFIIB (PDB ID: 1AIS)^70^, *P. woesei* TFIIB (PDB ID: 1D3U)^71^ and human TFIIB (PDB ID: 5IY6)^33^ as templates. The Brf1 homology block II (residues 435–510) was taken from yeast Brf1 (PDB ID: 1NGM)^40^ and helix H25 was extended based on the cryo-EM density. The N-terminal zinc ribbon domain of yeast TFIIB (PDB ID: 4BBR)^72^ was used as a template to build the N-terminal zinc ribbon domain of Brf1 and docked into the corresponding density.

To model the yeast C34 WH1 domain (residues 10–77), the solution structure of the mouse Rpc34 subunit (PDB ID: 2DK8) was used as a template to construct the yeast C34 WH1 structure. The homology model of the C34 WH2 domain (residues 78–158) was built using the structure of a MarR family transcriptional regulator (PDB ID: 3JW4) from *C. acetobutylicum*, the PSU-1 transcription regulator MarR (PDB ID: 3BRO) from *O. oeni* and the human solution structure of the WH domain in RPC34 (PDB ID: 2DK5) as templates.

The refined models for the core Pol III and the TFIIIB subunits (Brf1, TBP, and Bdp1) were separately rigid-body fitted into the four states of the complexes (ITC, OC, nOC, and the complex on the EC scaffold) and the models combined to assemble the full Pol III PIC models. The models were then subjected to real space refinement in the presence of secondary structure restraints using the PHENIX package^73^.

### Data availability

Cryo-EM density maps have been deposited in the Electron Microscopy Data Bank (EMDB) under accession numbers EMD-7530 (ITC), EMD-7531 (OC), EMD-7532 (nOC), EMD-7534 (iOC), EMD-7533 (the complex assembled on the EC scaffold). Model coordinates have been deposited in the Protein Data Bank (PDB) under accession numbers 6CNB (ITC), 6CNC (OC), 6CND (nOC), 6CNF (the complex assembled on the EC scaffold).

## Electronic supplementary material

Supplementary Information
