# Supplementary material for: Structural visualization of RNA polymerase III transcription machineries
Source: Cell Discov. 2018 Jul 31;4:40. doi: 10.1038/s41421-018-0044-z (PMC6066478; doi:10.1038/s41421-018-0044-z)

Supplementary information

Supplementary figure legends

Supp. Figure 1 Pol III factors used in the structural studies. a Protein gels showing purified Pol III factors. Pol III and TFIIIC were run on a 4-12% Bis-Tris gel and silver stained. Brf1N-TBPc-Brf1C and WT Bdp1 were run on a 12.5% Tris-Glycine gel and stained using Coomassie Blue. Bdp1 Δ355-372 was run on a 10% Tris-Glycine gel and stained using Coomassie Blue. Brf1N-TBPc-Brf1C and Bdp1 proteins are labeled with an asterisk. Molecular weight standards (kDa) are also shown. **b** Transcription assays using the purified Pol III factors. RNA standard sizes (number of nucleotide) are labeled to the right of the gel. The uncropped gel is shown in the lower panel. Boxed lanes were performed using mutants that were not reported in this paper.

Supp. Figure 2 Cryo-EM data processing of the Pol III ITC. a Representative negative stained raw micrograph. Scale bar = 500 nm. **b** Representative cryo-EM raw micrograph. Scale bar = 100 nm. **c** Refinement strategy (**Materials and Methods**). Euler angle distribution for the final 3D auto refinement is also shown. **d** FSC curves and estimated resolution using the 0.143 criteria following the gold-standard procedure implemented in RELION for the Pol III ITC.

Supp. Figure 3 Structural comparison of the Brf1/TBP/DNA complex. a Comparison with the human BRF2/TBP/DNA crystal structure (PDB ID: 4ROC)[1] reveals a similar position of the molecular pin in both Brf1 and BRF2. 4ROC is shown in gray, while our Brf1/TBP/DNA complex is shown using the same color scheme as in **Fig. 2**. **b**

Comparison with the yeast Brf1/TBP/DNA ternary complex crystal structure (PDB ID: 1NGM)[2] shows the homology block II of Brf1 adopts a similar location on TBP. 1NGM is shown in gray, while our Brf1/TBP/DNA complex is shown using the same color scheme as in **Fig. 2**.

Supp. Figure 4 Structural comparison of Bdp1. a Comparison with the human BRF2/TBP/BDP1/DNA crystal structure (PDB ID: 5N9G)[3] reveals a same position of the SANT domain of Bdp1. 5N9G is shown in gray, while our Bdp1/TBP/DNA complex is shown using the same color scheme as in **Fig. 2**. **b** The Bdp1 SANT domain and Brf1 C-terminus bind to a similar location on TBP as TFIIA (PDB ID: 1NVP)[4]. 1NVP is shown in gray, while our Bdp1/TBP/DNA complex is shown using the same color scheme as in **Fig. 2**.

Supp. Figure 5 Structural comparison of PICs of Pol I, II and III. a Comparison between Pol II and III PICs. The zinc ribbon and cyclin fold domains of TFIIB and TFIIIB occupy the polymerases at similar locations, suggesting a similar architecture between Pol II and III PICs. Polymerases were aligned for structural comparison. Pol II PIC (PDB ID: 5FYW)[5] is shown in gray, while Pol III PIC is depicted in the same color scheme as in **Fig. 1**. **b** Comparison between Pol I and III PICs. The zinc ribbon domains from Rrn7 and Brf1 contact the same location on the corresponding polymerases. On the contrary, the Rrn7 N-terminal cycling fold domain does not contact Pol I in the Pol I PIC, whereas the counterpart in Brf1 interacts with Pol III at the wall and protrusion. Pol I PIC (PDB ID:

5W65)[6] is shown in gray, while Pol III PIC is depicted in the same color scheme as in **Fig. 1**.

Supp. Figure 6 Putative density for the C-terminus of Brf1. The density is labeled with a blue circle. The ITC map is shown as transparency surface. The color scheme is the same as in **Fig. 1**.

Supp. Figure 7 Cryo-EM data processing of the Pol III OC. a Representative cryo-EM raw micrograph. Scale bar = 100 nm. **b** Refinement strategy (**Materials and Methods**). Euler angle distribution for the final 3D auto refinement is also shown. **c** FSC curves and estimated resolution using the 0.143 criteria following the gold-standard procedure implemented in RELION for the Pol III OC. Red, the full complex; blue, TFIIIB focused refinement. **d** Bottom view of the Pol III OC. Color scheme is the same as in **Fig. 1**.

Supp. Figure 8 Cryo-EM data processing of the Pol III nOC. a Representative cryo-EM raw micrograph. Scale bar = 100 nm. **b** Refinement strategy (**Materials and Methods**). Euler angle distribution for the final 3D auto refinement is also shown. **c** FSC curves and estimated resolution using the 0.143 criteria following the gold-standard procedure implemented in RELION for the Pol III nOC. **d** Bottom view of the Pol III nOC. Color scheme is the same as in **Fig. 1**.

Supp. Figure 9 Cryo-EM data processing of the Pol III iOC. a Representative cryo-EM raw micrograph. Scale bar = 100 nm. **b** Refinement strategy (**Materials and Methods**).

Euler angle distribution for the final 3D auto refinement is also shown. **c** FSC curves and estimated resolution using the 0.143 criteria following the gold-standard procedure implemented in RELION for the Pol III iOC. **d** Bottom view of the Pol III iOC. Color scheme is the same as in **Fig. 1**.

Supp. Figure 10 Cryo-EM data processing of the Pol III transcription complex assembled on the EC scaffold. a Representative cryo-EM raw micrograph. Scale bar = 100 nm. **b** Refinement strategy (**Materials and Methods**). Euler angle distribution for the final 3D auto refinement is also shown. **c** FSC curves and estimated resolution using the 0.143 criteria following the gold-standard procedure implemented in RELION for the Pol III transcription complex assembled on the EC scaffold. **d** Bottom view of the Pol III transcription complex assembled on the EC scaffold. Color scheme is the same as in **Fig. 1**.

Supp. Figure 11 TFIIIB aligned with the N-terminal cyclin fold domain in TFIIB. The Brf1 N-terminal cyclin fold domain was aligned with the N-terminal cyclin fold domain in TFIIB (PDB ID: 4BBR)[7]. This shows a rotational movement of the TFIIIB (colored) compared to the TFIIIB in the Pol III PIC (gray; polymerase was aligned with Pol II in 4BBR).

Supplementary references

1. Gouge, J. *et al.* Redox Signaling by the RNA Polymerase III TFIIB-Related Factor Brf2. *Cell* **163,** 1375–1387 (2015).

2. Juo, Z. S., Kassavetis, G. A., Wang, J., Geiduschek, E. P. & Sigler, P. B. Crystal structure of a transcription factor IIIB core interface ternary complex. *Nature* **422,** 534–539 (2003).
3. Gouge, J. *et al.* Molecular mechanisms of Bdp1 in TFIIIB assembly and RNA polymerase III transcription initiation. *Nature Communications* **8,** 5042 (2017).
4. Bleichenbacher, M., Tan, S. & Richmond, T. J. Novel interactions between the components of human and yeast TFIIA/TBP/DNA complexes. *Journal of Molecular Biology* **332,** 783–793 (2003).
5. Plaschka, C. *et al.* Transcription initiation complex structures elucidate DNA opening. *Nature* **533,** 353–358 (2016).
6. Han, Y. *et al.* Structural mechanism of ATP-independent transcription initiation by RNA polymerase I. *Elife* **6,** 753 (2017).
7. Sainsbury, S., Niesser, J. & Cramer, P. Structure and function of the initially transcribing RNA polymerase II-TFIIB complex. *Nature* **493,** 437–440 (2013).

Supp. Figure 1

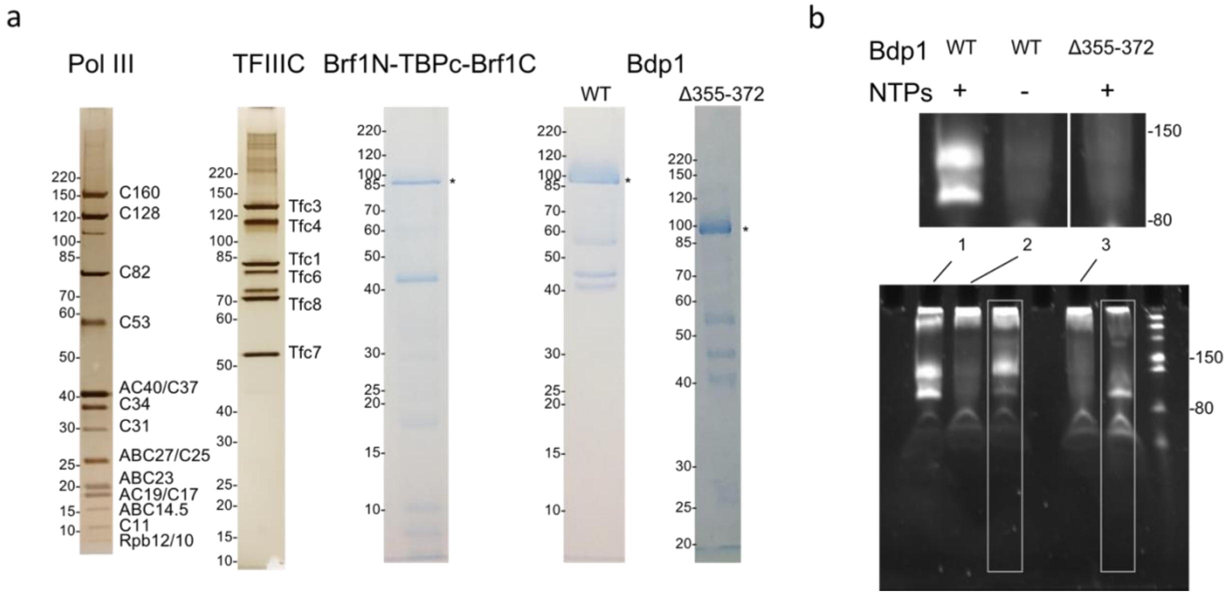

Supp. Figure 2

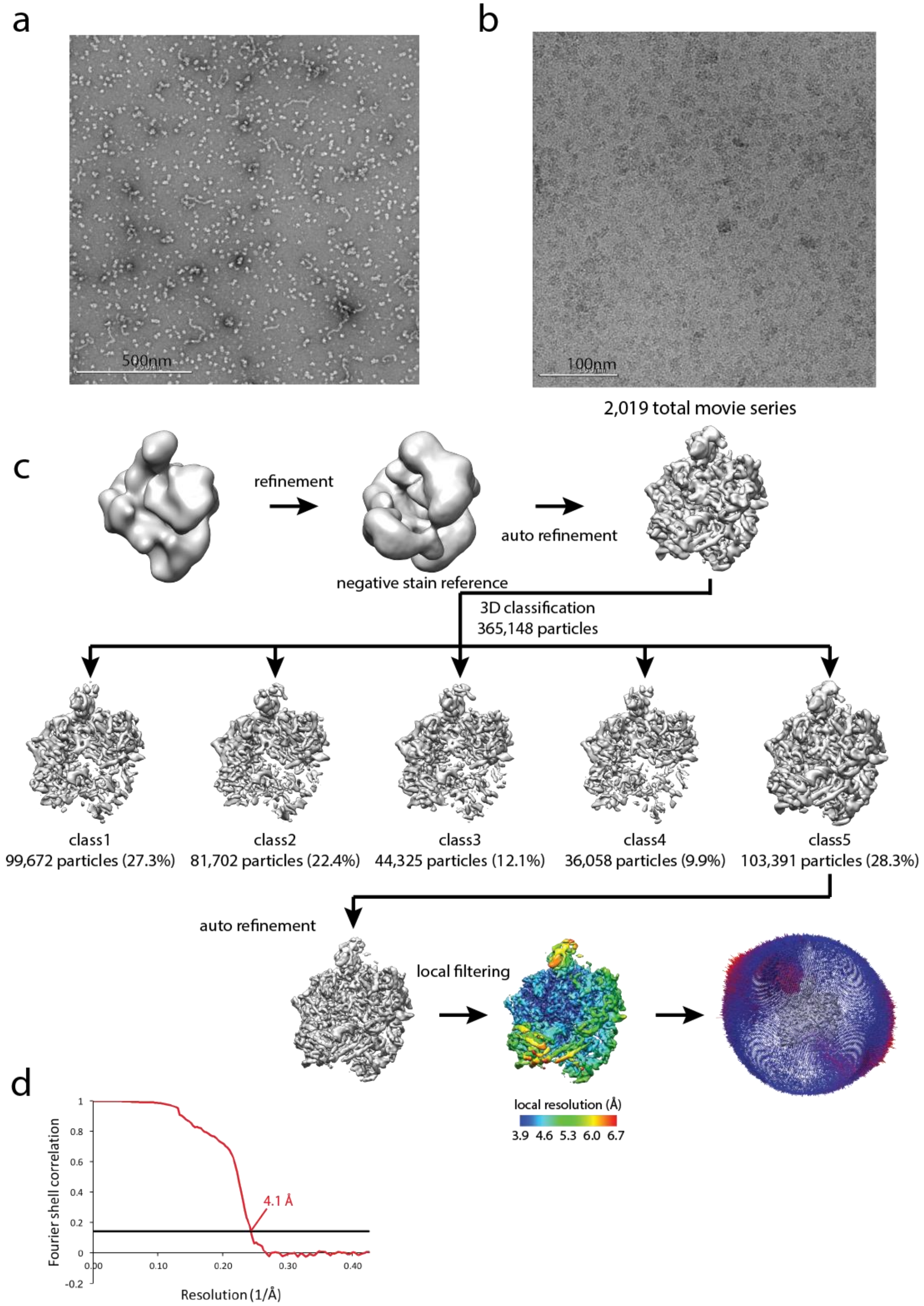

Supp. Figure 3

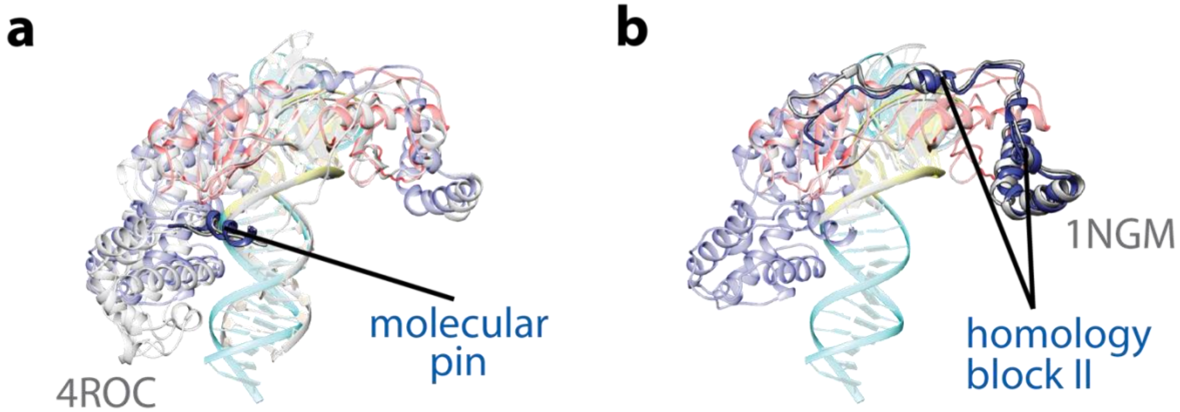

Supp. Figure 4

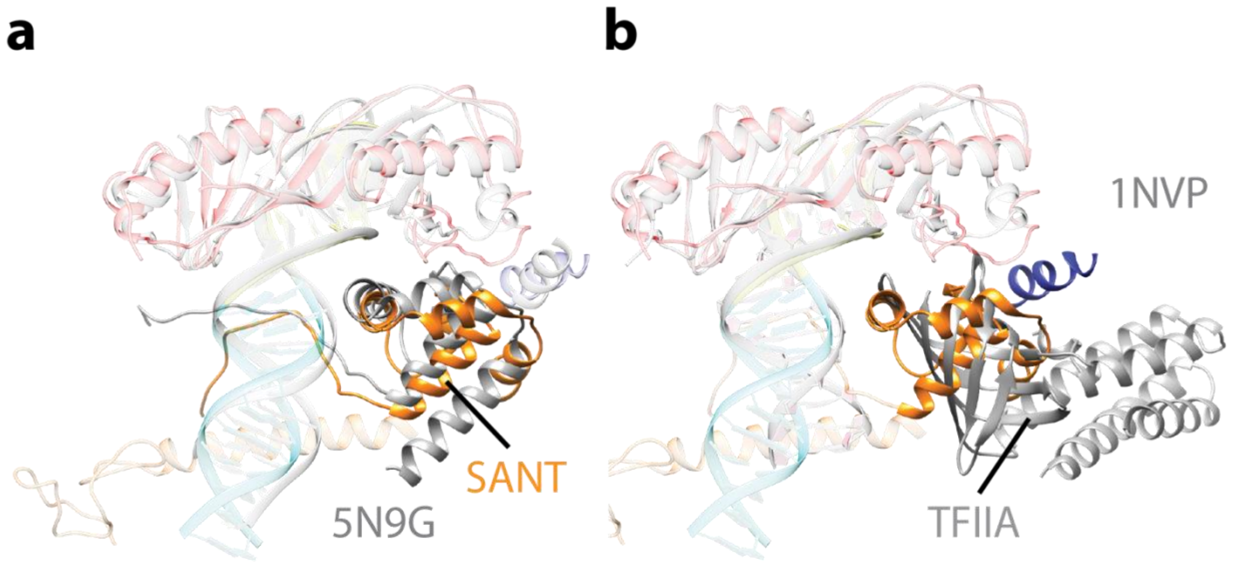

Supp. Figure 5

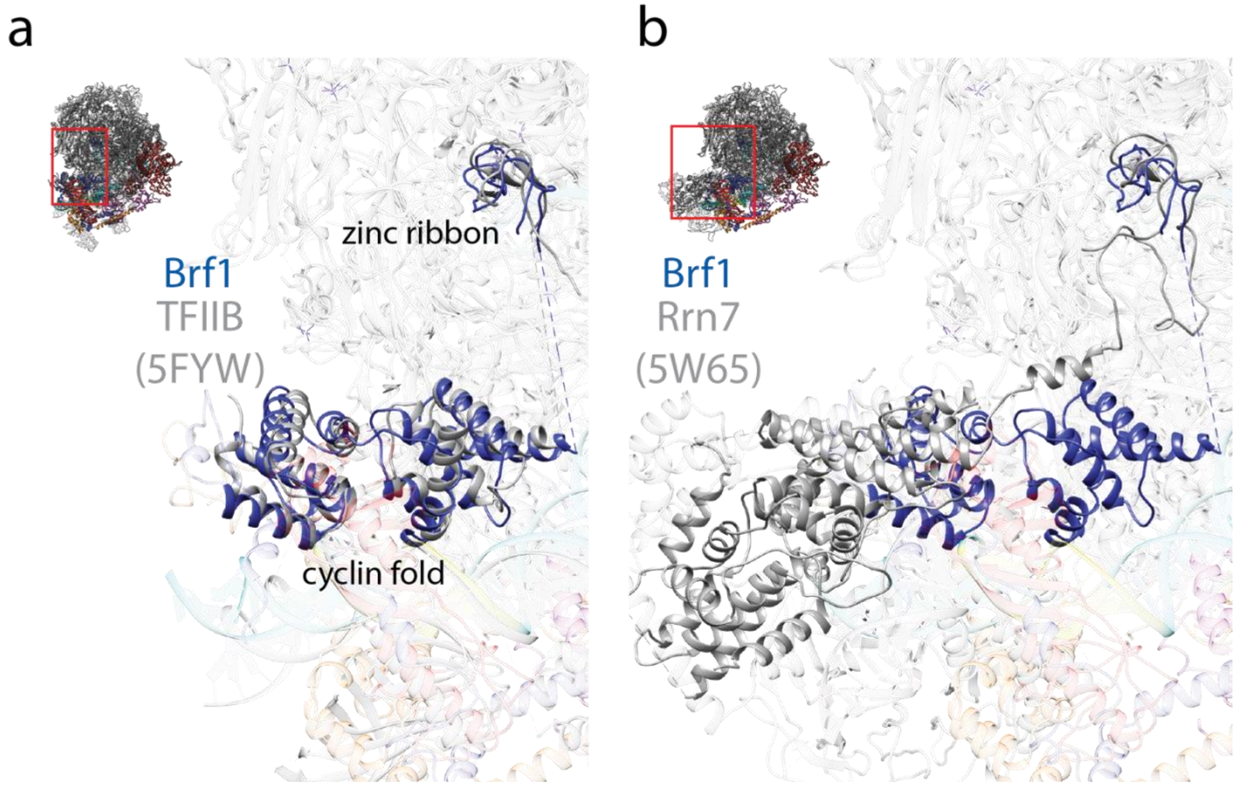

Supp. Figure 6

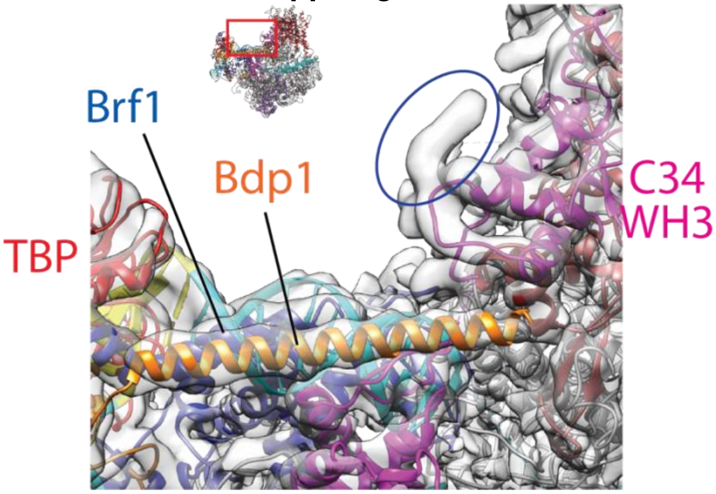

Supp. Figure 7

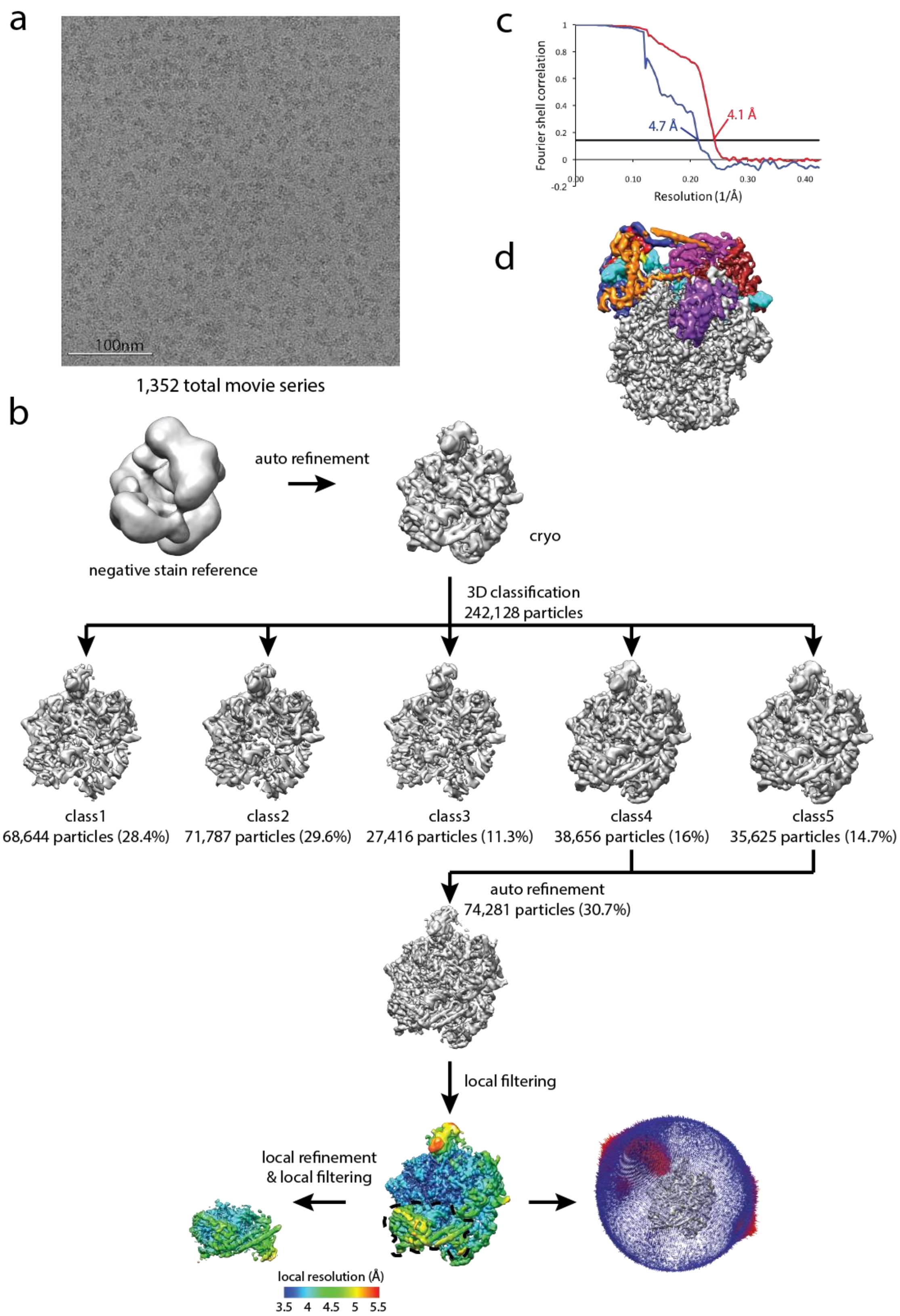

Supp. Figure 8

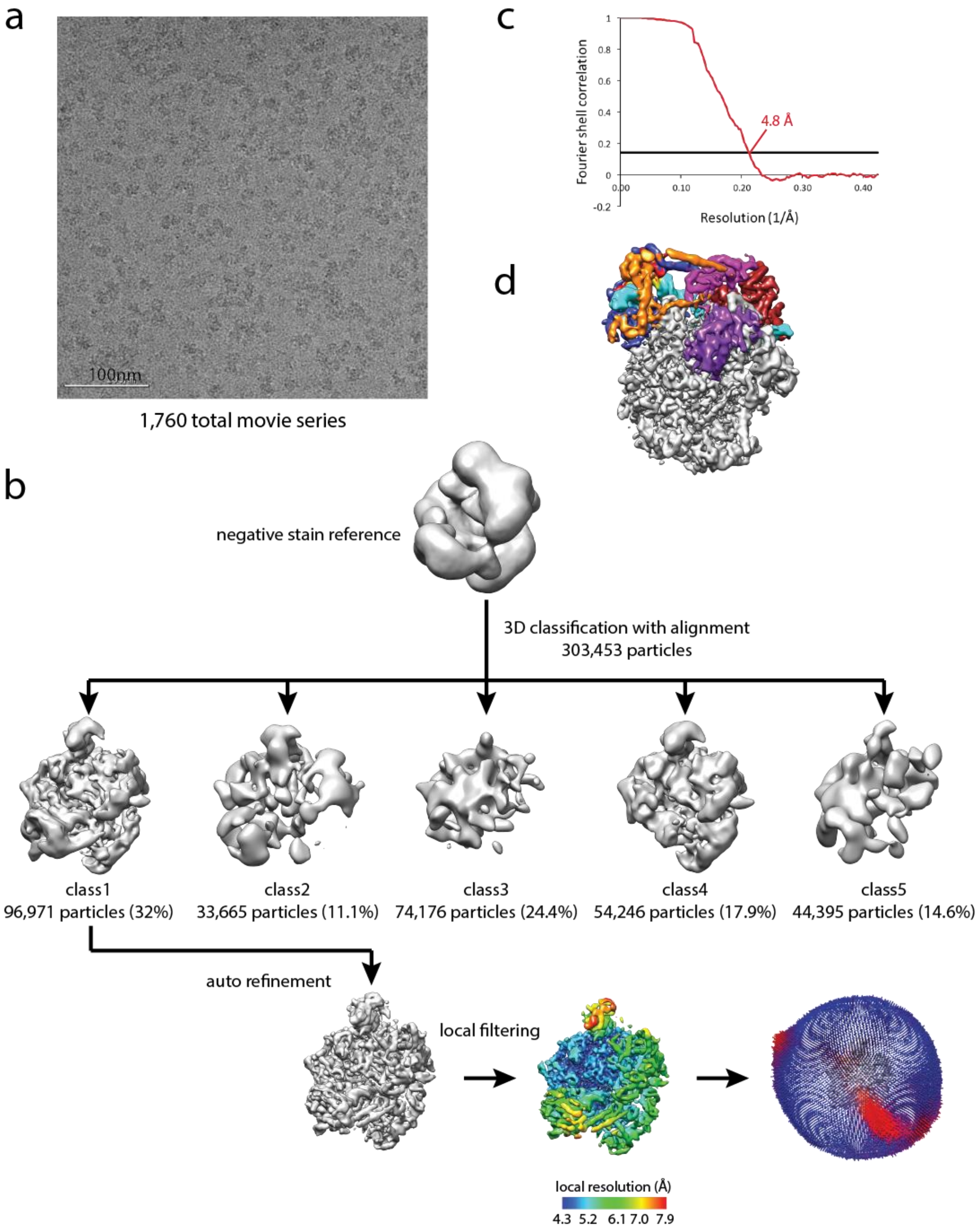

Supp. Figure 9

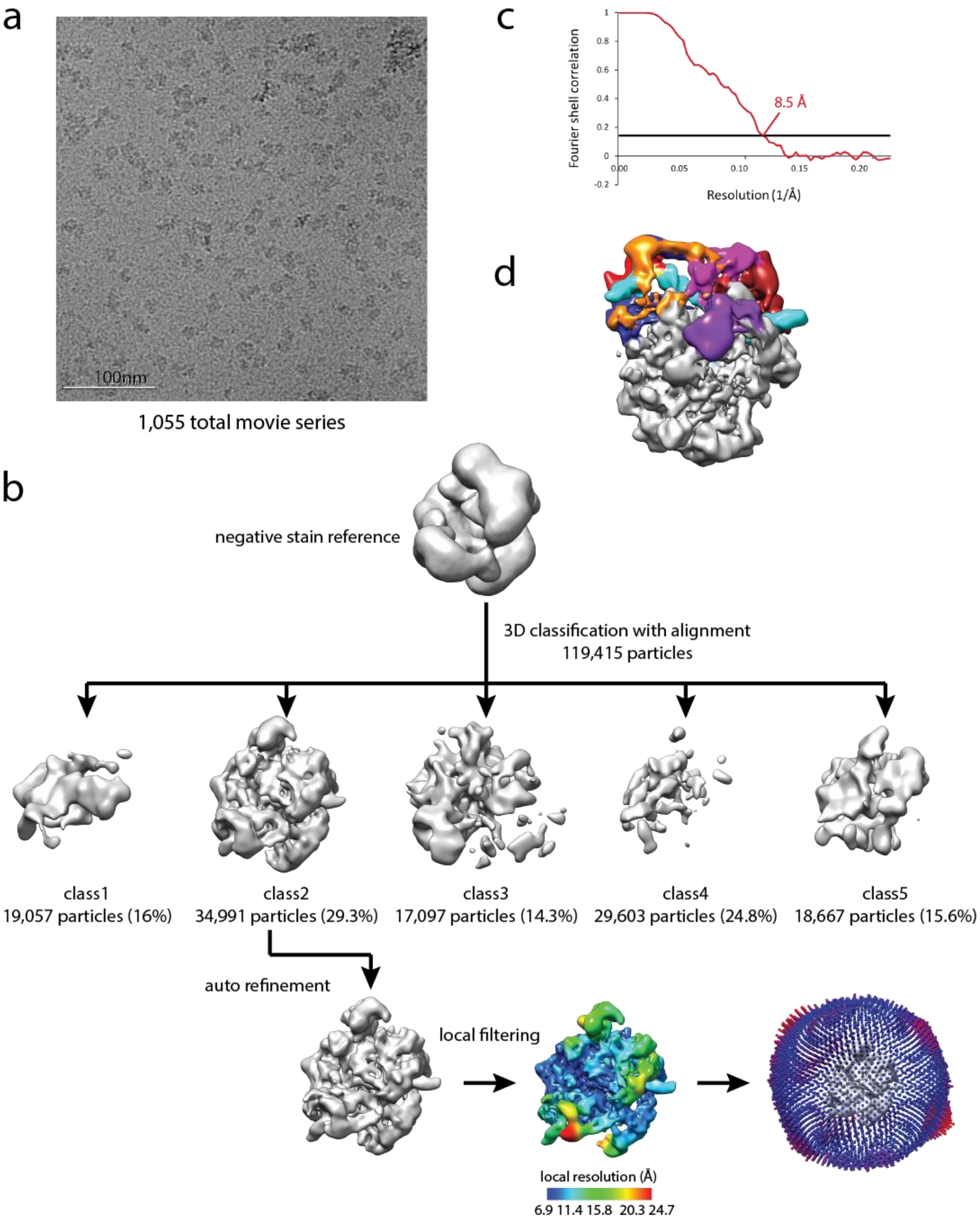

Supp. Figure 10

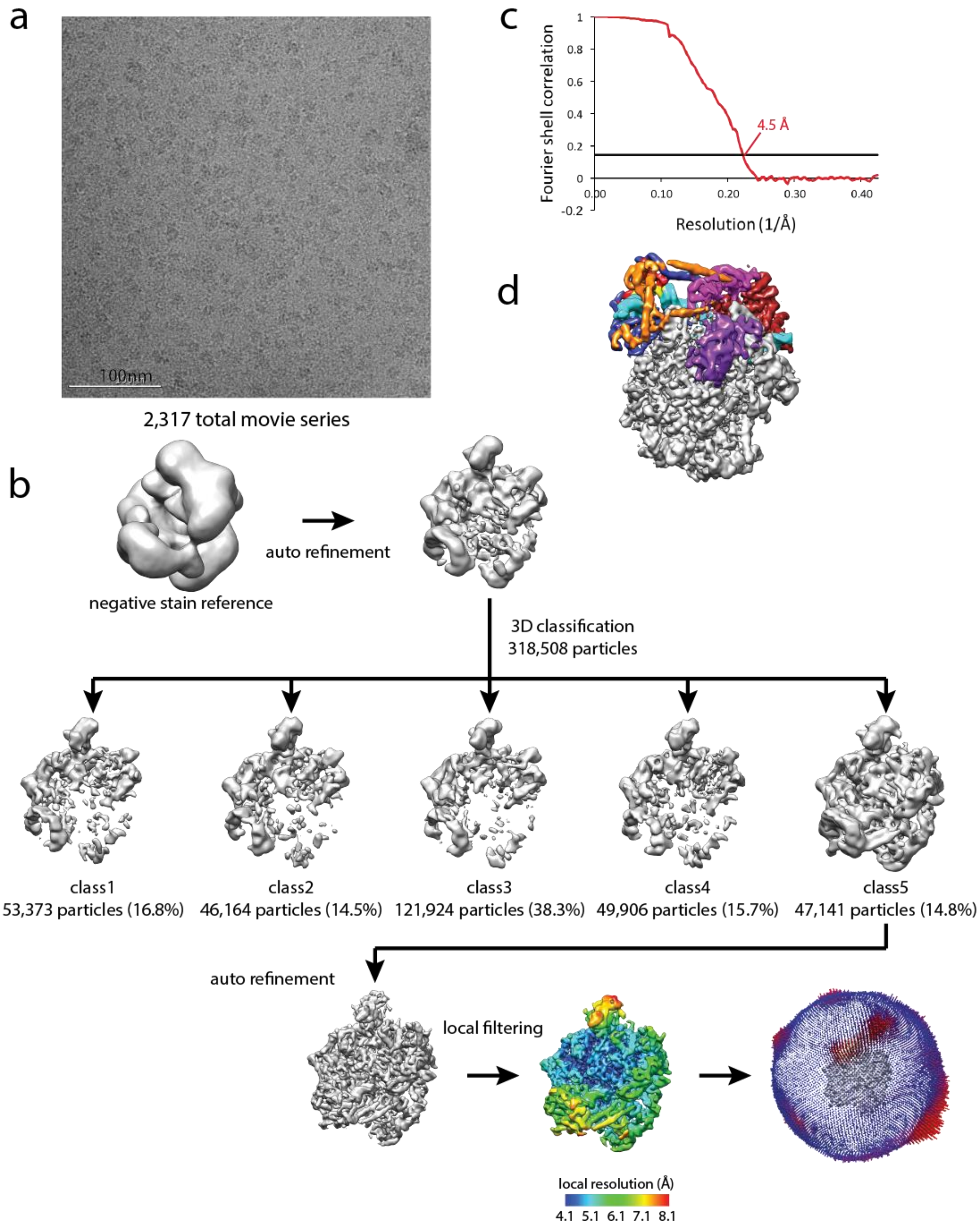

Supp. Figure 11

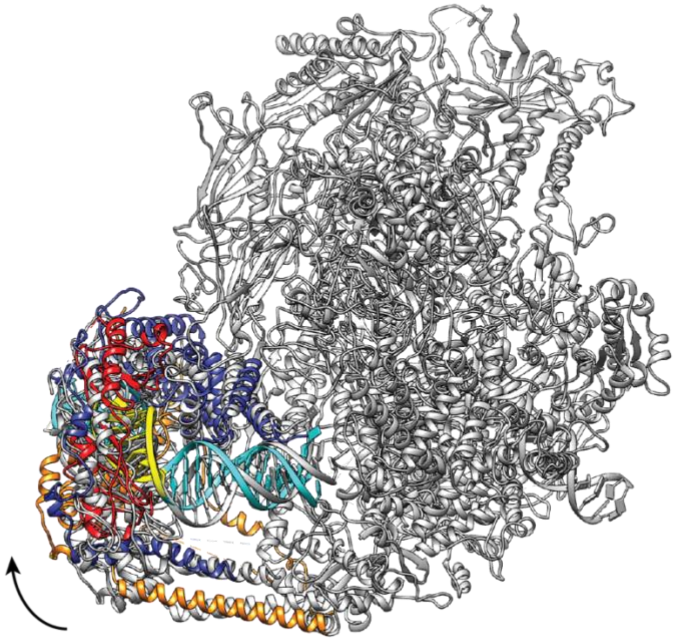

Supplement: Supplementary file 1 — Supplementary Information [file 41421_2018_44_MOESM1_ESM.pdf]
